# Obscurins: Goliaths and Davids Take over Non-Muscle Tissues

**DOI:** 10.1371/journal.pone.0088162

**Published:** 2014-02-06

**Authors:** Maegen A. Ackermann, Marey Shriver, Nicole A. Perry, Li-Yen R. Hu, Aikaterini Kontrogianni-Konstantopoulos

**Affiliations:** University of Maryland, School of Medicine, Department of Biochemistry and Molecular Biology, Baltimore, Maryland, United States of America; University of Palermo, Italy

## Abstract

Obscurins comprise a family of proteins originally identified in striated muscles, where they play essential roles in myofibrillogenesis, cytoskeletal organization, and Ca^2+^ homeostasis. They are encoded by the single *OBSCN* gene, and are composed of tandem adhesion domains and signaling motifs. To date, two giant obscurin isoforms have been described in detail that differ only at the extreme COOH-terminus; while obscurin-A (∼720 kDa) contains a non-modular COOH-terminus that harbors binding sites for the adaptor proteins ankyrins, obscurin-B (∼870 kDa) contains two COOH-terminal serine-threonine kinase domains preceded by adhesion motifs. Besides the two known giant obscurins, a thorough search of transcript databases suggests that complex alternative splicing of the obscurin transcript results in the generation of additional giant as well as small isoforms with molecular masses ranging between ∼50–970 kDa. These novel isoforms share common domains with the characterized isoforms, but also contain unique regions. Using a panel of highly specific antibodies directed against epitopes spanning the entire length of giant obscurins, we employed western blotting and immunohistochemistry to perform a systematic and comprehensive characterization of the expression profile of obscurins in muscle and non-muscle tissues. Our studies demonstrate for the first time that obscurins are not restricted to striated muscles, but are abundantly expressed in several tissues and organs including brain, skin, kidney, liver, spleen, and lung. While some obscurin isoforms are ubiquitously expressed, others are preferentially present in specific tissues and organs. Moreover, obscurins are present in select structures and cell types where they assume nuclear, cytosolic, and membrane distributions. Given the ubiquitous expression of some obscurins, along with the preferential expression of others, it becomes apparent that obscurins may play common and unique roles, respectively, in the regulation and maintenance of cell homeostasis in various tissues and organs throughout the body.

## Introduction

Obscurin was originally discovered about a decade ago during a yeast two-hybrid screen as a binding partner of the giant protein titin [1]. It was "baptized" obscurin by Young and colleagues because it was at first difficult to characterize due to its large size, low abundance, structural complexity, and insolubility in extracts of adult cardiac muscle. Today it is understood that obscurins are a family of proteins derived from the single *OBSCN* gene, which in humans spans >170 kb on chromosome 1q42.13.

**Figure 1:**
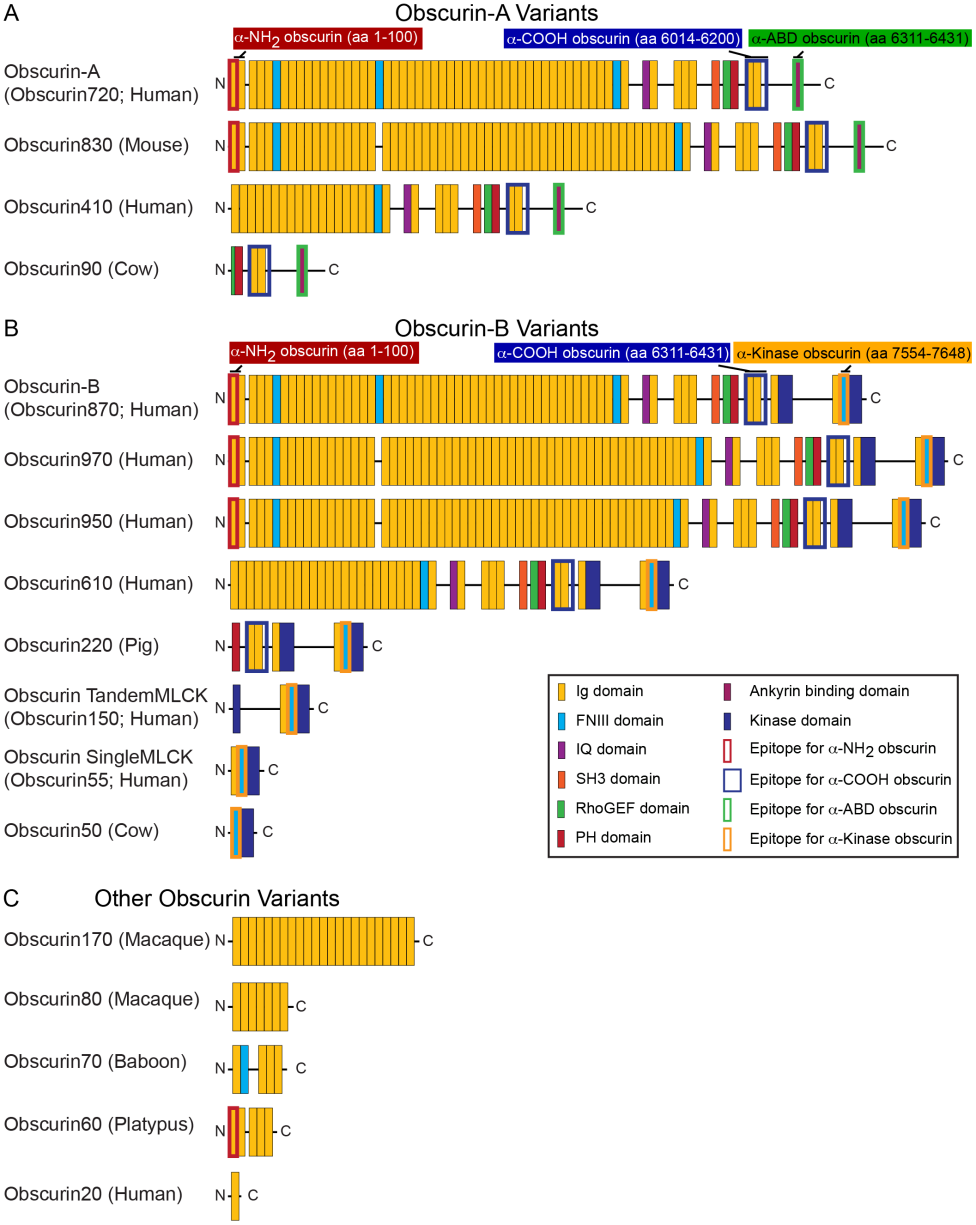
Mammalian obscurin variants. Domain architecture of up-to-date mammalian obscurin variants as listed in NCBI and Ensembl, illustrating their structural and signaling motifs (please see key for notations). Alternative splicing of the obscurin transcript results in several variants. (A) Obscurin-A-like isoforms, similar to prototypical obscurin-A, containing the non-modular COOH-terminus including the ankyrin-binding domain (ABD). (B) Obscurin-B-like isoforms containing one or both kinase domains, found in the COOH-terminus of obscurin-B. (C) Other splice variants containing sequences specific to neither obscurin-A-like nor obscurin-B-like proteins. The antigenic sequences used for the generation of the four obscurin antibodies are highlighted by the colored boxed regions (α-NH_2_ in red, α-COOH in blue, α-ABD in green, and α-kinase in yellow; the accession numbers that correspond to the amino acid coordinates of the antigenic sequences are stated in the Materials and Methods section).

Giant obscurins, namely obscurin-A and obscurin-B, share common domain architectures. They are composed of 68 immunoglobulin (Ig) and 3 fibronectin type-III (FNIII) adhesion domains, along with several signaling motifs, including an isoleucine-glutamine (IQ) calmodulin-binding motif, a src-homology 3 (SH3) domain, and tandem Rho-guanine nucleotide exchange factor (RhoGEF) and pleckstrin homology (PH) motifs. Obscurin-A (∼720 kDa; Fig. 1A) possesses a non-modular COOH-terminus of ∼400 amino acids that contains ankyrin binding domains (ABDs) as well as consensus phosphorylation motifs for ERK kinases [1]. Obscurin-B (∼870 kDa; Fig. 1B) lacks the non-modular COOH-terminal region found in obscurin-A, but includes two serine/threonine kinase (SK) domains that belong to the myosin light chain kinase (MLCK) subfamily, and are referred to as serine/threonine kinase 2 (SK2) and SK1 [2]. An Ig domain precedes SK2, while an Ig and an FNIII domain precede SK1. Alternative splicing of the obscurin precursor mRNA (pre-mRNA) also results in the expression of smaller kinase-containing obscurin isoforms, including tandem MLCK (∼120 kDa) that consists of at least part of SK2 and the full SK1 domain, and single MLCK that only contains SK1 (∼55 kDa) [2], [3], [4]; complete transcripts encoding the tandem and single obscurin kinase isoforms have yet to be identified.

Throughout the last decade, obscurins have been primarily and systematically studied in striated muscles [3], [5], [6]. Detailed immunofluorescence studies using cardiac and skeletal muscles and antibodies directed against different epitopes along the length of giant obscurins have demonstrated the presence of obscurins in diverse myofibrillar structures. Obscurins localize at the periphery of myofibrillar M-bands and Z-discs, the sarcolemma, the neuromuscular junction specific to skeletal muscle, and the intercalated disc unique to cardiac muscle [1], [7], [8], [9], [10]. The presence of multiple adhesion motifs in giant obscurins along with their distribution in multiple subcellular compartments in muscle cells allows them to provide binding sites for diverse proteins, and thus contribute to the functional integration of the sarcomeric cytoskeleton with internal membrane systems and the sarcolemma. For a comprehensive review on the ligands and functions of obscurins, we refer the reader to recent reviews [5], [6].

Recent studies from our laboratory have documented the presence of obscurins in normal breast epithelial cells, too, where they exhibit nuclear, cytosolic, and membrane distributions, and contribute to the regulation of cell survival [11].

To date, little work has been done to examine the expression profile and functional properties of obscurins in non-muscle tissues. However, earlier studies have suggested the presence of obscurin transcripts at low levels in non-muscle tissues, including brain, liver, kidney, and pancreas [12]. Herein, we systematically examined the expression profile and subcellular localization of obscurins in muscle and non-muscle tissues and organs of small rodents, including heart, tibialis anterior (TA), quadriceps, soleus, and diaphragm muscles as well as brain, skin, kidney, liver, spleen, and lung. Using western blot analysis and a panel of antibodies recognizing epitopes along the length of giant obscurins, we show for the first time the presence of numerous obscurin isoforms that range in size from ∼50–970 kDa in muscle and non-muscle tissues. While some obscurin isoforms are ubiquitously expressed, others are tissue specific. Moreover, using immunohistochemistry, we show that obscurins localize to distinct structures and populations of cells within different tissues and organs, where they exhibit nuclear, cytosolic, or membrane distributions. Our studies are the first to provide a comprehensive characterization of the expression profile and subcellular distribution of obscurins in muscle and non-muscle tissues and organs, implicating them in the regulation and maintenance of diverse cellular processes in mammals.

## Results and Discussion

### Obscurins are a Multifaceted Family of Proteins

Since the original identification of the human *OBSCN* gene in 2001 [1], the most well studied obscurin isoforms have been the canonical obscurins, A and B. In addition, two smaller isoforms containing either the tandem or single kinase domains present in the extreme COOH-terminus of obscurin-B are understood to exist [2], [3], [4]; however, their complete domain architectures have yet to be elucidated.

Importantly, the extensive technological advancements of the last decade have shed light on the complexity of many mammalian and non-mammalian transcriptomes, revealing the presence of additional transcripts originating from the single *OBSCN* gene (see Table 1 and Table S1 for an up-to-date list of isoforms that are encoded by these transcripts). For the purposes of this study, we have only included mammalian obscurin transcripts possessing both a start and stop codon, and either partial or complete 5′ and 3′ untranslated regions (UTRs). We have denoted each additional transcript according to the calculated molecular weight of the protein it encodes, and categorized the isoforms into three subpopulations: i. isoforms similar to obscurin-A (“obscurin-A-like”), containing the non-modular COOH-terminus (Fig. 1A), ii. isoforms similar to obscurin-B (“obscurin-B-like”), possessing at least one kinase domain (Fig. 1B), and iii. additional isoforms originating from the *OBSCN* gene but not resembling the COOH-terminus of either obscurin-A or -B (Fig. 1C).

**Table 1:** List of mammalian obscurin isoforms.

Notation	Species	Mol Wt (kDa)	Contains the epitope for:							Transcript Database (accession #)
			α-NH_2_	α-COOH		α-ABD			α-Kinase	
***Obscurin-A-like Isoforms***										
Obscurin-A	Human	720	+		+		+	–		NCBI (NM_052843)^# ˆ^
Obscurin-A	Mouse	815	+		+		+	–		NCBI (NM_199152)#
Obscurin830	Mouse	830	+		+		+	–		Ensembl (ENSMUSP00000020732)^?^
Obscurin410	Human	410	–		+		+	–		Ensembl (ENSP00000355670)
***Obscurin-B-like Isoforms***										
Obscurin-B	Human	870	+		+		–	+		NCBI (NM_001098623)#
Obscurin-B	Mouse	875	+		+		–	+		NCBI (NM_001171512)#
Obscurin970	Human	970	+		+		–	+		NCBI (NM_001271223)^?^
Obscurin950	Human	950	+		+		–	+		Ensembl (ENSP00000455507)
Obscurin610	Human	610	–		+		–	+		Ensembl (ENSP00000355668)
TandemMLCK	Human	150*/120**	–		–		–	+		
SingleMLCK	Human	70–55**	–		–		–	+		
***Additional Obscurin Isoforms***										
Obscruin20	Human	20	–		–		–	–		NCBI (BC114382)

*According to personal communication with Dr. Mark Russell.

**Referenced in Hu and Kontrogianni-Konstantopoulos 2013.

# Also found in Ensembl.

ˆAlso found in Vega.

To date, canonical obscurin-A transcripts have been identified in three species: human (coding for a protein of ∼720 kDa), mouse (∼815 kDa) and opossum (∼730 kDa). Variations in the molecular weights of obscurin-A across species correspond to differences in the number of Ig domains within the NH_2_-terminus and the middle of each protein. Alternative splicing of the obscurin transcript leads to additional obscurin-A-like isoforms. For instance, mouse obscurin-830 (∼830 kDa) differs from mouse obscurin-A by 15 kDa due to the addition of two Ig domains at the NH_2_-terminus of the protein. Similarly, alternative splicing of the human obscurin transcript results in an obscurin-A-like isoform, obscurin-410 (∼410 kDa) that lacks twenty-eight Ig and two FNIII domains.

Furthermore, canonical obscurin-B has been identified in six mammalian species, including human (∼870 kDa), mouse (∼875 kDa), horse (∼840 kDa), dolphin (∼840 kDa), megabat (∼840 kDa), and orangutan (∼835 kDa). Similar to obscurin-A, the variations in the calculated molecular weights among obscurin-B homologues correspond to differences in the number of Ig domains at the NH_2_-terminus of the respective proteins. The human *OBSCN* gene gives rise to three additional obscurin-B-like isoforms: obscurin-970, obscurin-950, and obscurin-610. Obscurin-970 and obscurin-950 are composed of all the adhesion and signaling domains found in obscurin-B but also possess novel Ig domains present in the NH_2_-terminus of the protein; obscurin-970 has eleven additional Ig domains, while obscurin-950 retains eight of those novel domains. Obscurin-610 is a smaller version of obscurin-B, as it lacks the first twenty-two Ig domains and two FNIII domains of the giant isoform, but retains the remaining adhesion and signaling domains present in the COOH-terminus of obscurin-B. An additional, smaller isoform, obscurin-20, has been identified in the human transcriptome; this transcript encodes 166 amino acid residues including Ig17 and the first ∼50 residues of the succeeding FNIII domain.

Additional isoforms have been identified in other mammalian systems (Table S1). A small obscurin-A-like isoform, with a predicted molecular weight of ∼90 kDa, has been identified in the cow genome. Obscurin-90 starts in the middle of the RhoGEF domain and includes the PH and ABD motifs present in the COOH-terminus of obscurin-A. Two additional obscurin-B-like isoforms have been identified in the pig and cow transcriptomes, obscurin-220 and obscurin-50, respectively. Obscurin-220 is a truncated version of obscurin-B, beginning at the PH domain and retaining both Ser/Thr kinase domains. On the other hand, obscurin-50 contains only the second kinase domain, SKI, along with the FNIII domain preceding it. Interestingly, obscurin-50 may correspond to the single MLCK isoform that has been described in the human transcriptome, although the precise molecular identity of the latter has yet to be characterized. Additional smaller obscurin isoforms have been identified in the macaque (obscurin-170 and obscurin-80), baboon (obscurin-70), and platypus (obscurin-60) transcriptomes. Each of these contains Ig and FNIII domains found within the NH_2_-terminus of the larger obscurins; however, they lack the signaling domains present in the COOH-terminus of either obscurin-A- or -B-like isoforms.

**Figure 2:**
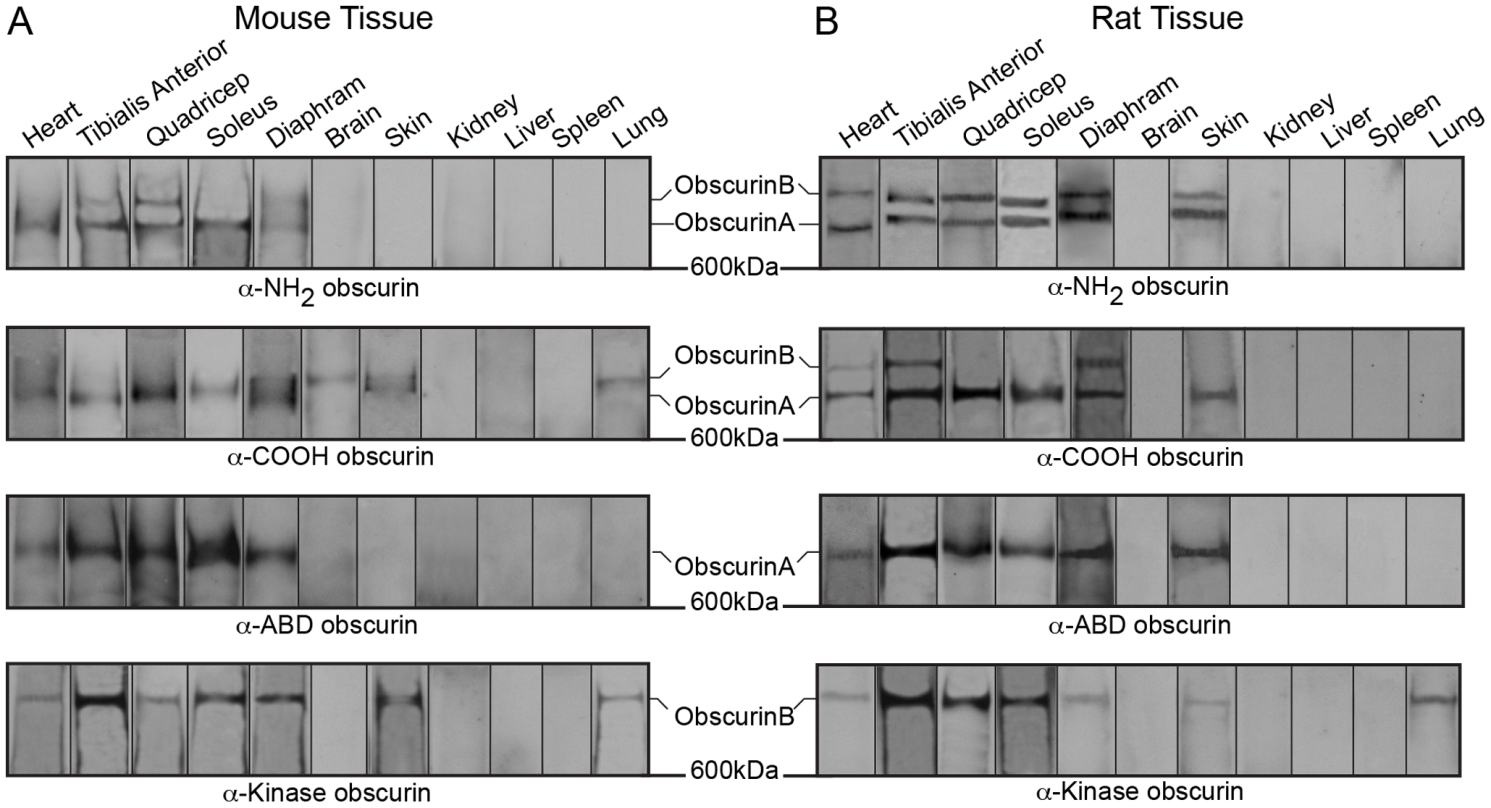
Expression of giant obscurins in rodent tissues and organs. Western blot analysis of 70 µg of protein homogenates prepared from adult mouse (A) and rat (B) tissues were probed with four antibodies to obscurins: α-NH_2_, α-COOH, α-ABD, and α-Kinase (the epitope for each antibody is noted in Figure 1). The blots have been cut at ∼600 kDa to focus on the giant forms of obscurin, and representative lanes from multiple experiments are shown. In agreement with previously published data, giant obscurin-A and -B are consistently identified in both cardiac and skeletal muscles of mouse and rat origin using any of the four obscurin antibodies. Notably, they are also present in select non-muscle tissues including brain, skin, and lung. Similar protein content per lane was ensured with a GAPDH load control.

**Figure 3:**
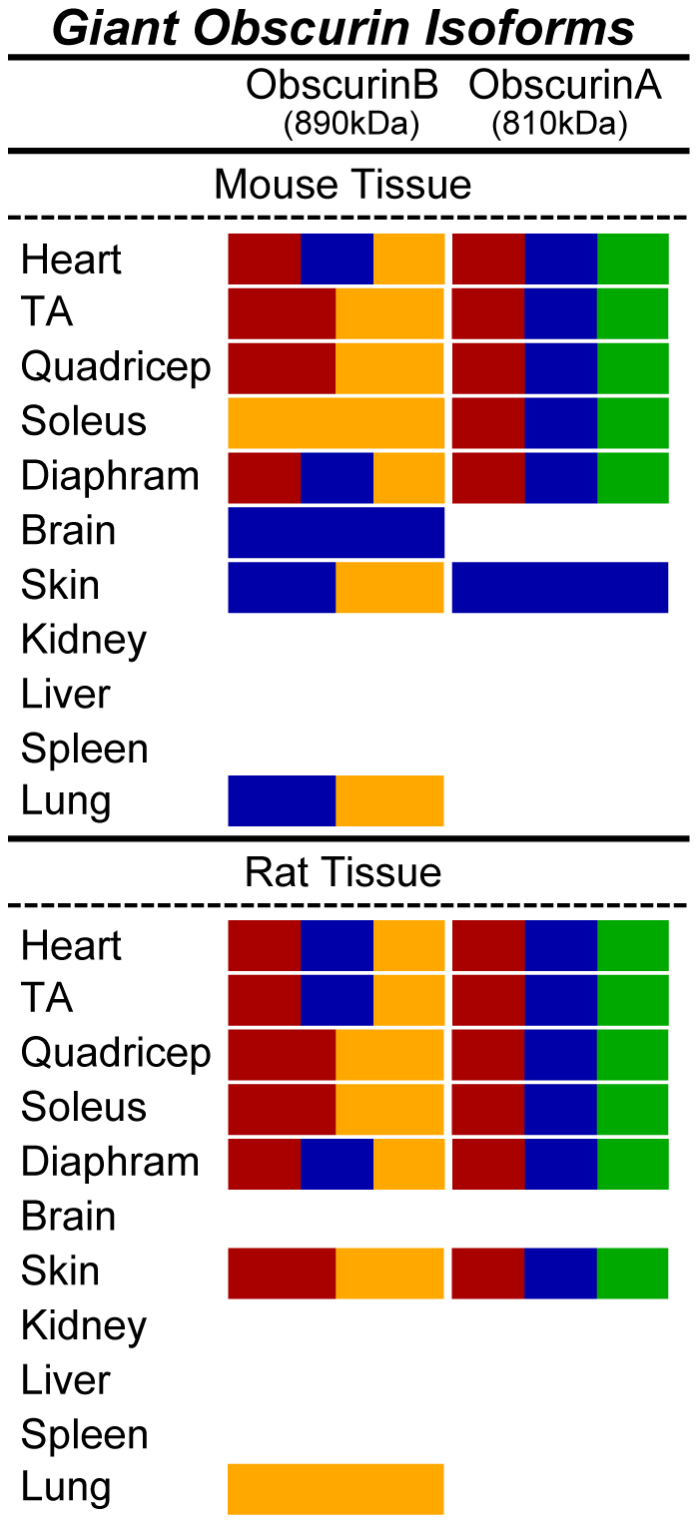
Epitopes present in giant obscurins. The ability of each of the four obscurin antibodies (α-NH_2_ in red, α-COOH in blue, α-ABD in green, and α-Kinase in yellow) to recognize giant obscurins (>60 kDa) is depicted for each murine tissue and organ.

**Figure 4:**
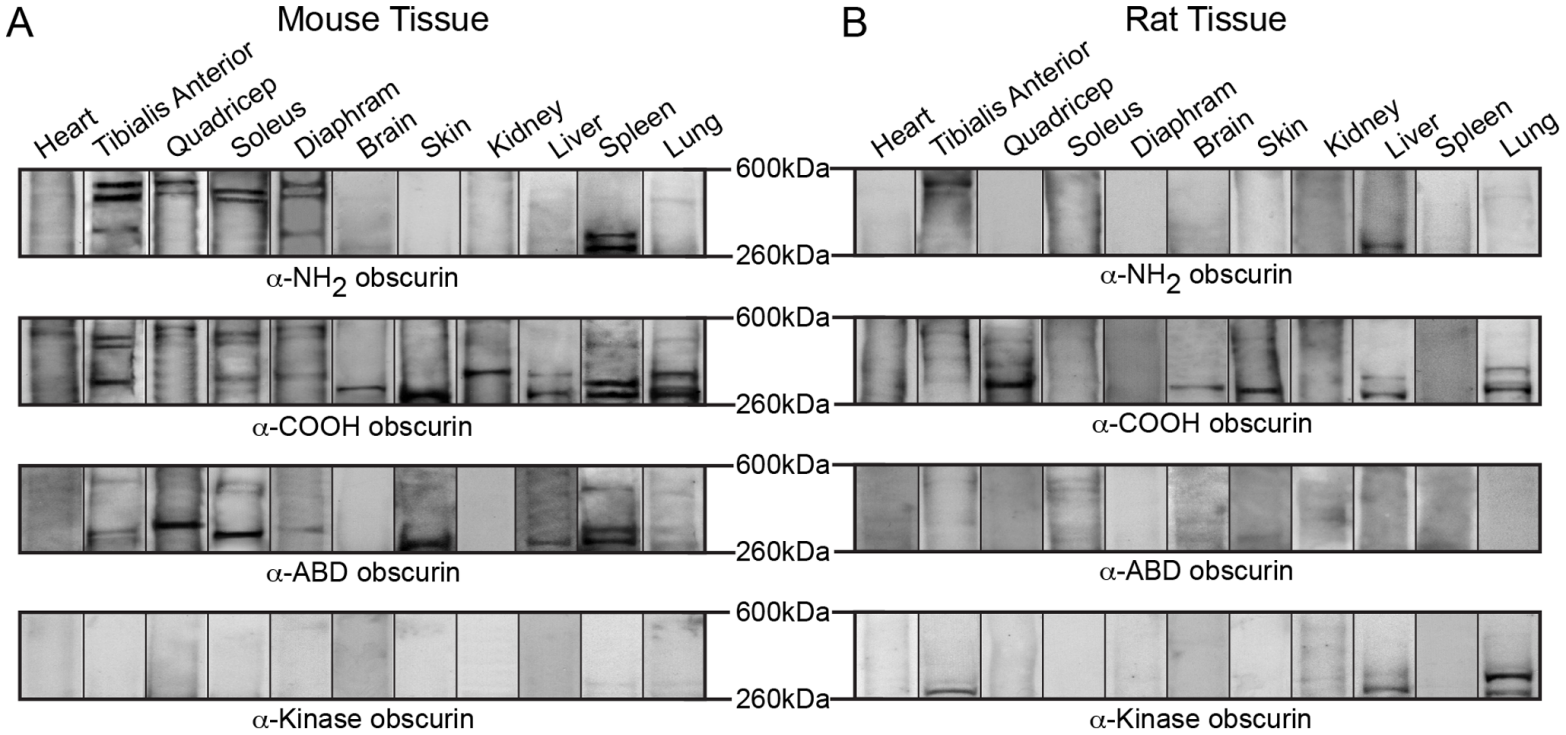
Expression of intermediate obscurins in rodent tissues and organs. Western blot analysis of 70 µg of protein homogenates prepared from various adult mouse (A) and rat (B) tissues were probed with antibodies specific to obscurins. As before, probing for GAPDH ensured equal loading. The blots have been cut to include intermediate obscurins, ranging in size between ∼260–600 kDa. Each lane is a representative image from multiple replicates.

**Figure 5:**
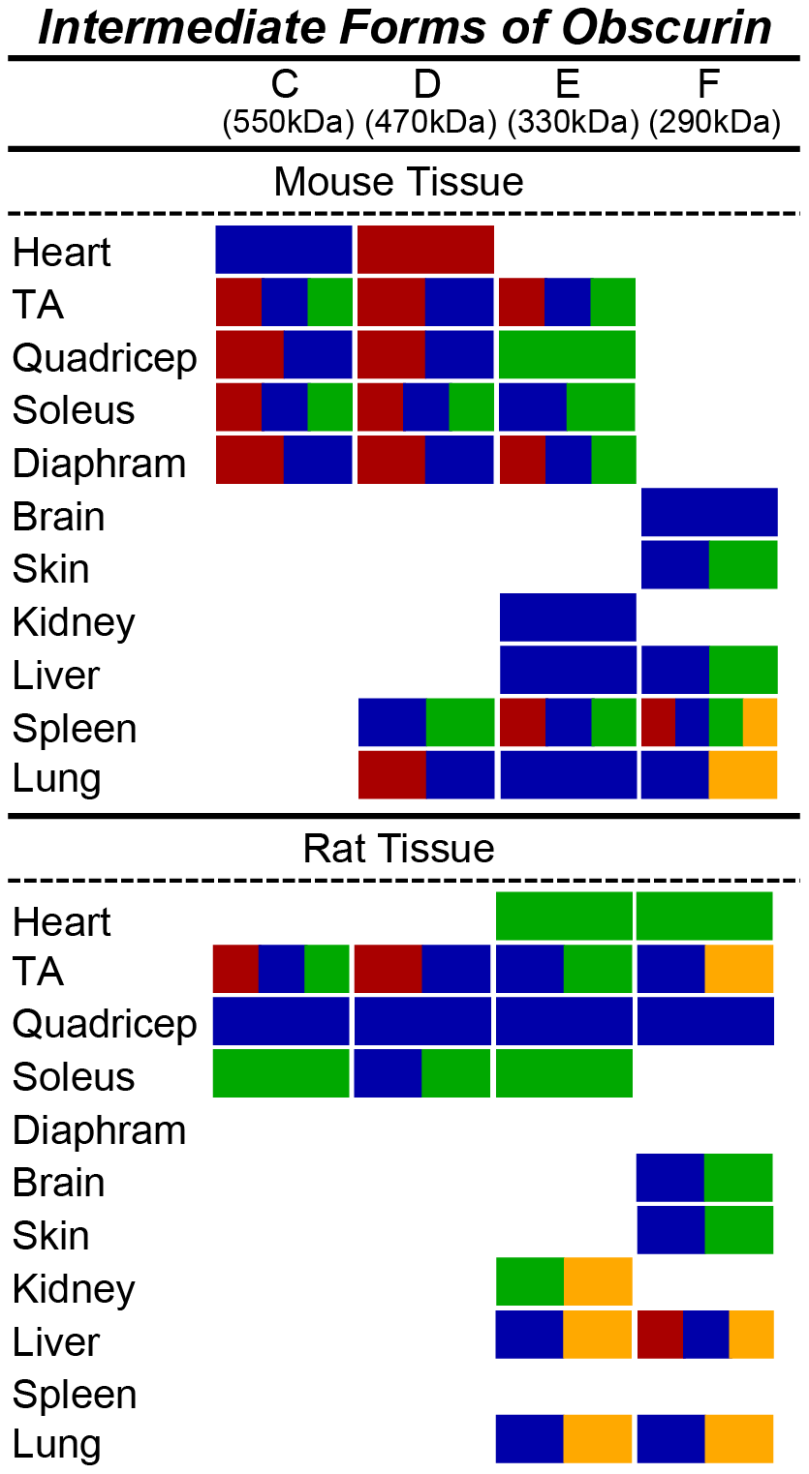
Epitopes present in intermediate obscurins. The ability of each of the four obscurin antibodies (α-NH_2_ in red, α-COOH in blue, α-ABD in green, and α-Kinase in yellow) to recognize intermediate obscurins (∼260–600 kDa) is noted for each murine tissue and organ.

**Figure 6:**
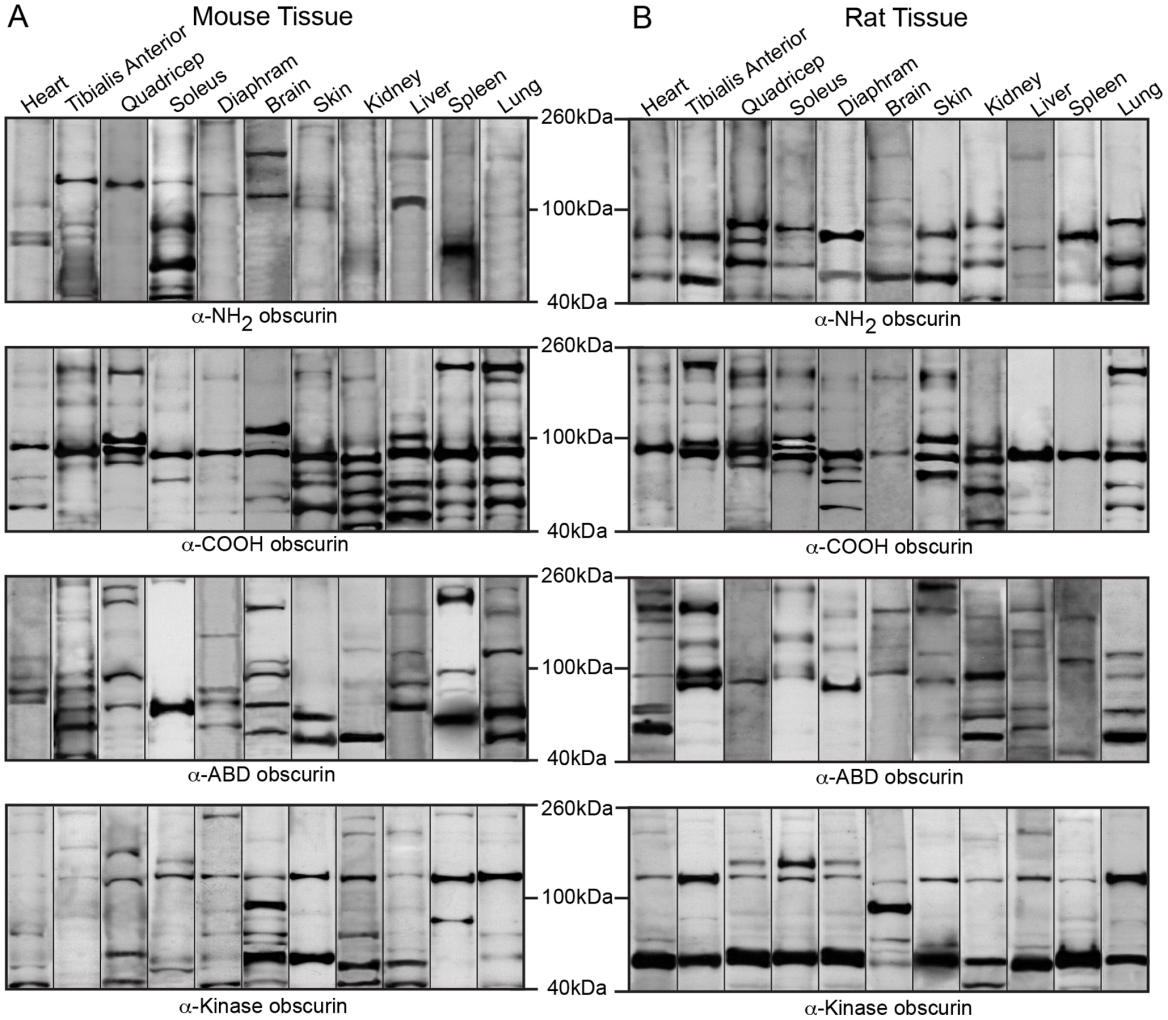
Expression of small obscurins in rodent tissues and organs. Western blot analysis of 70 µg of protein homogenates prepared from various adult mouse (A) and rat (B) tissues were probed with antibodies specific to obscurins and a GAPDH loading control. The blots have been cut to show small obscurins with molecular weights of ∼40–260 kDa. A representative blot for each tissue is shown in every lane.

**Figure 7:**
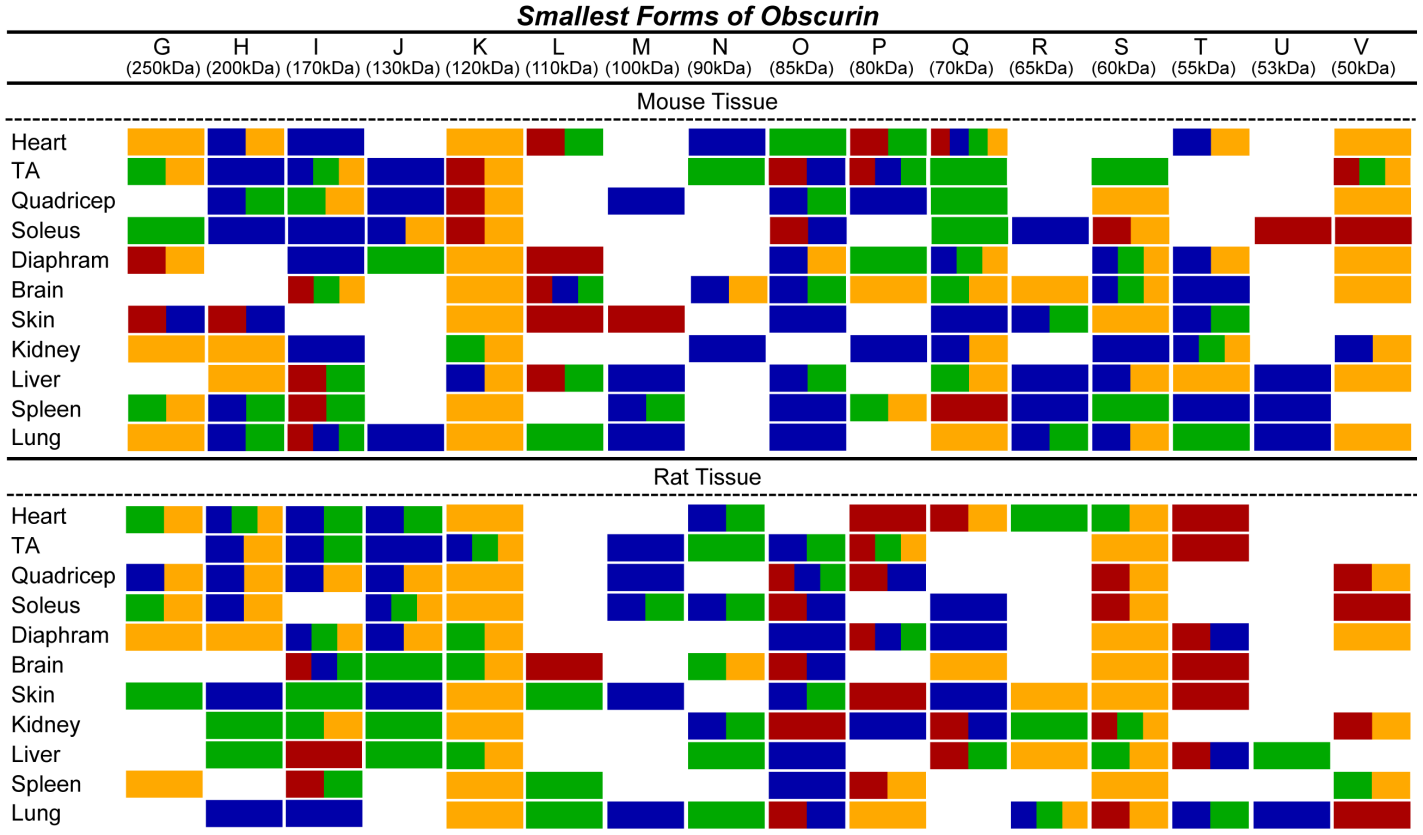
Epitopes present in small obscurins. The ability of each of the four obscurin antibodies (α-NH_2_ in red, α-COOH in blue, α-ABD in green, and α-Kinase in yellow) to recognize small obscurins (∼40–260 kDa) is depicted for each murine tissue and organ.

The expression pattern of the different obscurin isoforms has yet to be elucidated. We have therefore commenced characterization of the expression of obscurins in mouse and rat tissues by performing western blot analysis using lysates from various striated muscles (heart, TA, quadriceps, soleus, and diaphragm), as well as non-muscle tissues (brain, skin, kidney, liver, spleen, and lung) (Figs. 2–7). To this end, we used 70 µg of total protein from each tissue and probed for obscurins using a panel of four different antibodies recognizing epitopes along the length of the canonical giant isoforms. Specifically, the α-NH_2_, α-COOH, α-ABD, and α-Kinase antibodies are directed against the first Ig domain (Ig1) [3], the two Ig domains following the RhoGEF/PH module (Ig65/Ig66) [9], the ABD specific to obscurin-A-like isoforms [13], and the FNIII domain preceding SK1 found only in obscurin-B-like isoforms [10], respectively (Fig. 1). We identified several obscurins ranging in size from ∼50–970 kDa. Some of the observed immunoreactive bands may correspond to proteins encoded by the transcripts that have been deposited in sequence repositories (as discussed in the three preceding paragraphs), while others may represent novel isoforms whose transcripts have yet to be identified. In addition, we probed all tissue lysates (70 µg) for glyceraldehyde 3-phosphate dehydrogenase (GAPDH) to show equal loading (Fig. S1). Minor differences in the levels of GAPDH most likely reflect the differential expression of the enzyme across tissues [14], [15].

In accordance with previous studies, mouse and rat striated muscles express obscurin-A- and B-like isoforms (Figs. 2 and 3) [1], [2], [10], [16]. Due to the complexity of the obscurin family and the inability to precisely determine the molecular weights of the immunoreactive bands detected at the top of the polyacrylamide gel, it is unclear if these correspond to the canonical obscurins A (∼720 kDa) and B (∼870 kDa) or to the recently reported larger isoforms (i.e. the obscurin-A-like isoform, obscurin-830, or the obscurin-B-like isoforms, obscurin-970 and obscurin-950). The differential expression of giant obscurins among striated muscles is not clearly understood. For instance, we observe variations in the mobility of the largest obscurin-A-like and obscurin-B-like isoforms among tested muscles (Fig. 2A–B, lanes 1–5); this suggests that either select giant obscurins are preferentially expressed in particular striated muscles, or that the same isoforms undergo post-translational modifications specific to each muscle. Interestingly, non-muscle organs also express giant obscurins. In particular, mouse and rat skin express both obscurin-A-like and obscurin-B-like giant isoforms, while mouse brain and lung, and rat lung, contain an obscurin-B-like giant isoform. The presence of an obscurin-B-like isoform in mouse brain but not rat brain is surprising, and may be explained either by the inaccessibility or absence of the respective obscurin antigen in rat brain lysates or by the differential expression of obscurin-B-like isoforms between mouse and rat tissues.

In the various muscle and non-muscle tissues and organs examined, we observed at least four additional obscurin isoforms with intermediate molecular weights, ranging between ∼290–550 kDa (Figs. 4 and 5). Immunoreactive bands of ∼550, ∼470, ∼330, and ∼290 kDa were observed in mouse and rat striated muscles. Non-muscle tissues also contain different combinations of these immunoreactive bands. Mouse and rat brain, skin, liver and lung express a ∼290 kDa isoform, while mouse and rat kidney, liver, and lung contain a ∼330 kDa isoform. Mouse spleen, but not rat spleen, also expresses isoforms of ∼290 kDa, ∼330 kDa and ∼470 kDa. In addition, mouse lung contains a ∼470 kDa isoform, however, this was not found in rat lung.

Interestingly, in addition to the giant and intermediate obscurins, we detected several smaller immunoreactive bands ranging in size between ∼50–250 kDa. In particular, using our panel of antibodies, we observed at least sixteen immunoreactive bands within both muscle and non-muscle tissues and organs; the particular isoforms expressed and their relative abundance are unique to each tissue (Figs. 6 and 7). For simplicity, we will focus our description on the immunoreactive bands that possibly correspond to the mammalian isoforms identified in the different transcript databases; however, a complete list of immunoreactive bands within this molecular weight range is provided in Figure 7. A ∼200 kDa immunoreactive band that was predominantly detected in mouse and rat striated muscles as well as skin, kidney, lung, and liver may correspond to obscurin-220, a smaller form of obscurin-B (Fig. 1B). Similarly, the ∼120 kDa protein that is expressed in all samples may represent the tandem-MLCK obscurin isoform (Fig. 1B) [10]. Moreover, recent work from our group has shown that the single-MLCK obscurin isoform undergoes glycosylation, which alters its apparent molecular weight from ∼50 to ∼70 kDa [10]. Therefore, it is likely that the immunoreactive bands in the ∼50–70 kDa range represent differentially glycosylated forms of the single MLCK obscurin protein (Fig. 1B). It is also possible that the ∼90 kDa isoform, preferentially expressed in select striated muscles (e.g. heart and TA) and non-muscle tissues (e.g. brain and kidney), corresponds to obscurin-90, which is composed of the COOH-terminus of obscurin-A (Fig. 1A). Lastly, the ∼60 kDa immunoreactive band in rat muscle and non-muscle tissues may correspond to obscurin-60, which carries the extreme NH_2_-terminus of giant obscurins (Fig. 1C).

Taken together, these results provide for the first time biochemical evidence that the *Obscn* genes of both mouse and rat can give rise to multiple isoforms with diverse molecular weights, ranging between ∼50 and ∼970 kDa. Consistent with this, the majority of the tandem adhesion and signaling domains present in the murine *Obscn* genes are encoded by individual exons, have complementary splice sites, and preserve the open reading frame [2], [4]. The expression of multiple obscurin isoforms by a single tissue is not without precedent. Specifically, immunoblot analysis of protein lysates prepared from murine striated muscles using antibodies to the common COOH-terminus of giant obscurins indicated the presence of immunoreactive bands of ∼100 and ∼150 kDa [3]; notably, we also detect a ∼100 kDa isoform in select murine striated muscles (e.g. TA, quadriceps, and soleus) and non-muscle organs (e.g. skin, liver, spleen, and lung). Moreover, a recent study demonstrated the presence of ∼110 and ∼120 kDa obscurin isoforms in nuclear lysates prepared from human breast epithelial cells; these isoforms contained the RhoGEF and kinase domains, respectively [11]. Accordingly, we have also observed immunoreactive bands of ∼110 kDa in mouse brain and of ∼120 kDa in all murine striated muscles and non-muscle organs sampled. Although the presence of multiple obscurin isoforms with distinct structural compositions is the most plausible explanation of the different immunoreactive bands detected in the tissues and organs that we examined, we cannot preclude the possibility that at least some of the observed bands may represent degradation products of larger obscurins. Detailed molecular characterization of the different obscurin transcripts is therefore needed before we are able to assign novel obscurin isoforms with certainty. Nevertheless, the extensive alternative splicing that obscurin transcripts undergo represents an effective mechanism for generating distinct obscurin proteins with different structural and regulatory properties that may modulate select cellular processes.

### Subcellular Distribution of Obscurins in Striated Muscles

**Figure 8:**
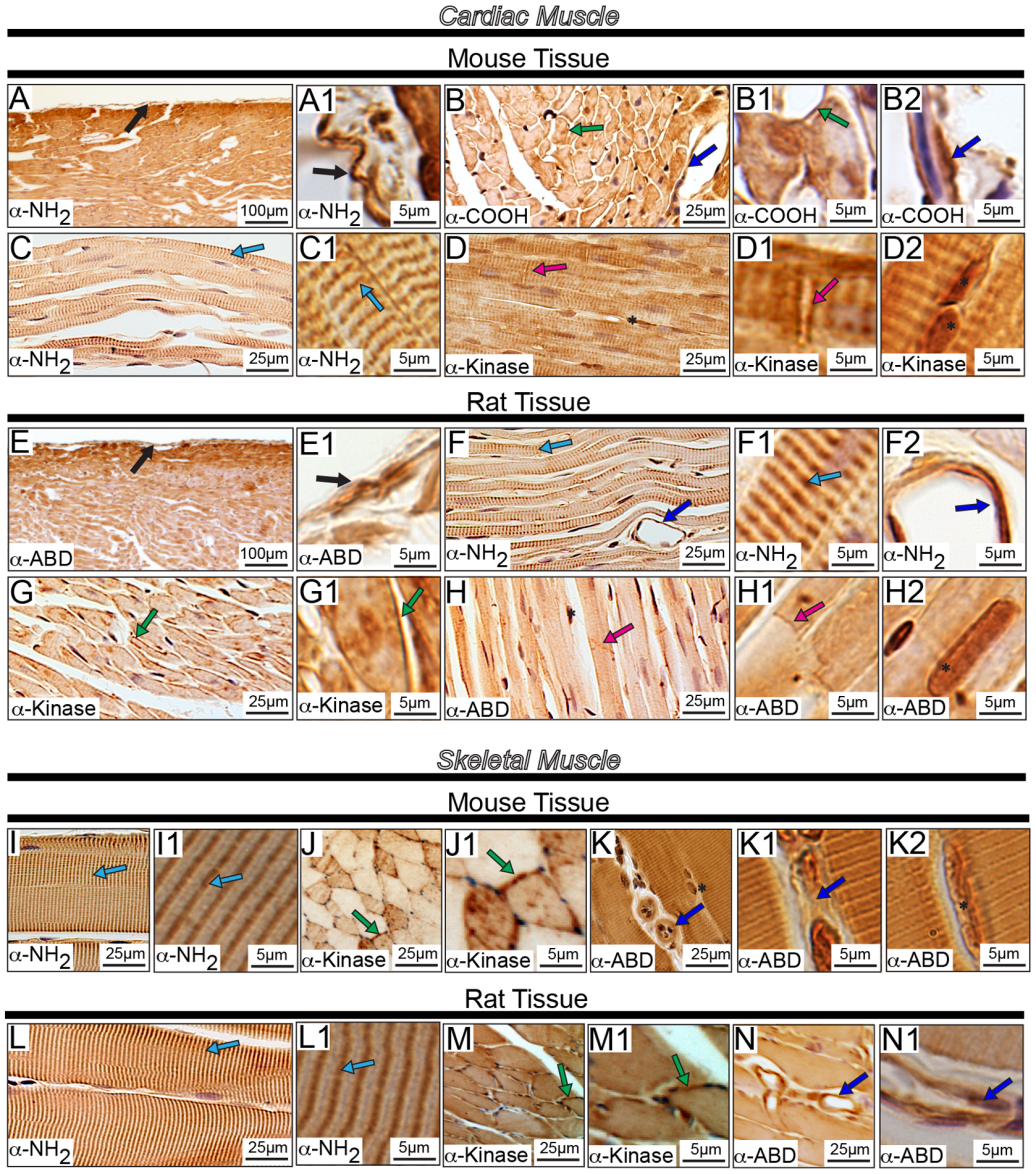
Localization of obscurins in rodent striated muscles. Adult mouse and rat heart (A-H2) and tibialis anterior (I-N1) muscle sections were analyzed by immunohistochemistry using antibodies specific for obscurins. In accordance with previous studies, obscurins exhibited a striated pattern in both cardiac and skeletal muscles of mouse and rat origin. **Cardiac** **Tissue**: Obscurins reside in the mouse and rat epicardium (A-A1 and E-E1, respectively; black arrows). In the myocardial layer, obscurins are found at the sarcolemma (B-B1 and G-G1, mouse and rat tissues, respectively; green arrows) and in sarcomeric striations (C-C1 and F-F1, mouse and rat tissues, respectively; light blue arrows). Interestingly, intercalated disks (pink arrows) and the nuclei of cardiomyocytes (black asterisk) are also labeled in both mouse (D-D1) and rat (H-H2) tissues. In addition, obscurins are found within the cells lining the vasculature throughout the heart (B and B2, and F and F2, mouse and rat tissues, respectively; dark blue arrows). **Skeletal Muscle** **Tissue:** Similar to earlier observations, obscurins localize to myofibrillar striations (light blue arrows) of both mouse (I-I1) and rat (L-L1) tissues. Obscurins are also present at the sarcolemma (J-J1 and M-M1, mouse and rat tissues, respectively; green arrows) and the nuclei of mouse, but not rat, skeletal muscle using the α-ABD antibody (K and K2, black asterisk). Moreover, obscurins are found within the cells lining the walls of the vasculature (dark blue arrow) in both mouse (K-K1) and rat (N-N1) tissue. Images are shown at multiple magnifications to highlight the various immunopositive structures. Scale bars are included in each panel for reference; for details please refer to the relevant section in the Materials and Methods.

The localization of obscurins in striated muscles has been extensively studied under the confocal [1], [13], [17], [18] and electron [3], [17], [19] microscopes. Using immunohistochemical methods and antibodies to epitopes spanning the length of giant obscurins (Fig. 1 and Table S2), we herein show that in the murine heart, obscurins localize in the outer layer of the heart, called the epicardium (Fig. 8 A–A1 and E-E1, black arrows), and to the myocardium (Fig. 8 B–H). In the myocardium, obscurins are present at the sarcolemma (Fig. 8 B–B1 and G-G1, green arrows) and the intercalated disk (Fig. 8 D–D1 and H-H1, pink arrows) as well as in striations (Fig. 8 C–C1 and F-F1, light blue arrows). Similar to cardiomyocytes, obscurins localize in sarcomeric striations (Fig. 8 I–I1 and L–L1, light blue arrows) and the sarcolemma (Fig. 8, J–J1 and M-M1, green arrows) in murine skeletal myofibers. These results are consistent with previous studies reporting that obscurins localize at the periphery of myofibrillar M-bands and Z-disks of skeletal myofibers and cardiomyocytes [1], [3], [8], [20], [21], as well as the intercalated disk of cardiocytes [10]. Importantly, the presence of obscurins in different cellular structures was observed with more than one antibody, and in some instances (e.g. the epicardium and the sarcomeric striations) with all four antibodies, including those specific to obscurin-A-like (α-ABD) and obscurin-B-like (α-kinase) epitopes. These observations suggest that both obscurin-A-like and obscurin-B-like isoforms may co-exist within the same subcellular compartment.

We also observed nuclear localization (Fig. 8 D and D2, H and H2, and K and K2, black asterisk) of obscurins in both mouse and rat cardiac and skeletal muscles with select antibodies. Although this was an unexpected finding, the functional relevance of which needs to be further examined, it is not without a precedent. Our group has recently shown that two small obscurin isoforms of ∼110 and ∼120 kDa that contain the RhoGEF and kinase domains, respectively, are selectively enriched in the nuclei of breast epithelial cells and cardiomyocytes [10], [11]. Moreover, while the presence of obscurins in sarcomeric striations has been well documented [1], [3], [13], [21], their localization at the sarcolemma and the intercalated disk has only recently been postulated [7], [9], [10]. Consistent with this, Carlsson and colleagues reported that obscurins are enriched at the neuromuscular junction [7], while Hu and Kontrogianni-Konstantopoulos documented that a small obscurin kinase isoform (∼50–70 kDa) localizes extracellularly in striated muscles [10], [11]. In addition, recent studies from our laboratory have indicated the presence of a small obscurin isoform that contains the tandem RhoGEF and PH motifs, and preferentially concentrates at the intercalated disk (unpublished observations). In accordance with these observations, Perry and colleagues demonstrated the presence of obscurins at the cell membrane and the Golgi apparatus of breast epithelial cells [11].

In addition to the various distributions of obscurins in murine striated muscles, they are also abundantly expressed in the vasculature (Fig. 8 B and B2, F and F2, K–K1, and N–N1, dark blue arrows). All four antibodies stained the vasculature in striated muscle, which is consistent with a previous report indicating the presence of obscurins in endothelial cells that make up the lining of capillaries in skeletal muscles [7], although neither the molecular identity of these obscurin isoforms nor their role was investigated.

### Subcellular Distribution of Obscurins in the Brain

**Figure 9:**
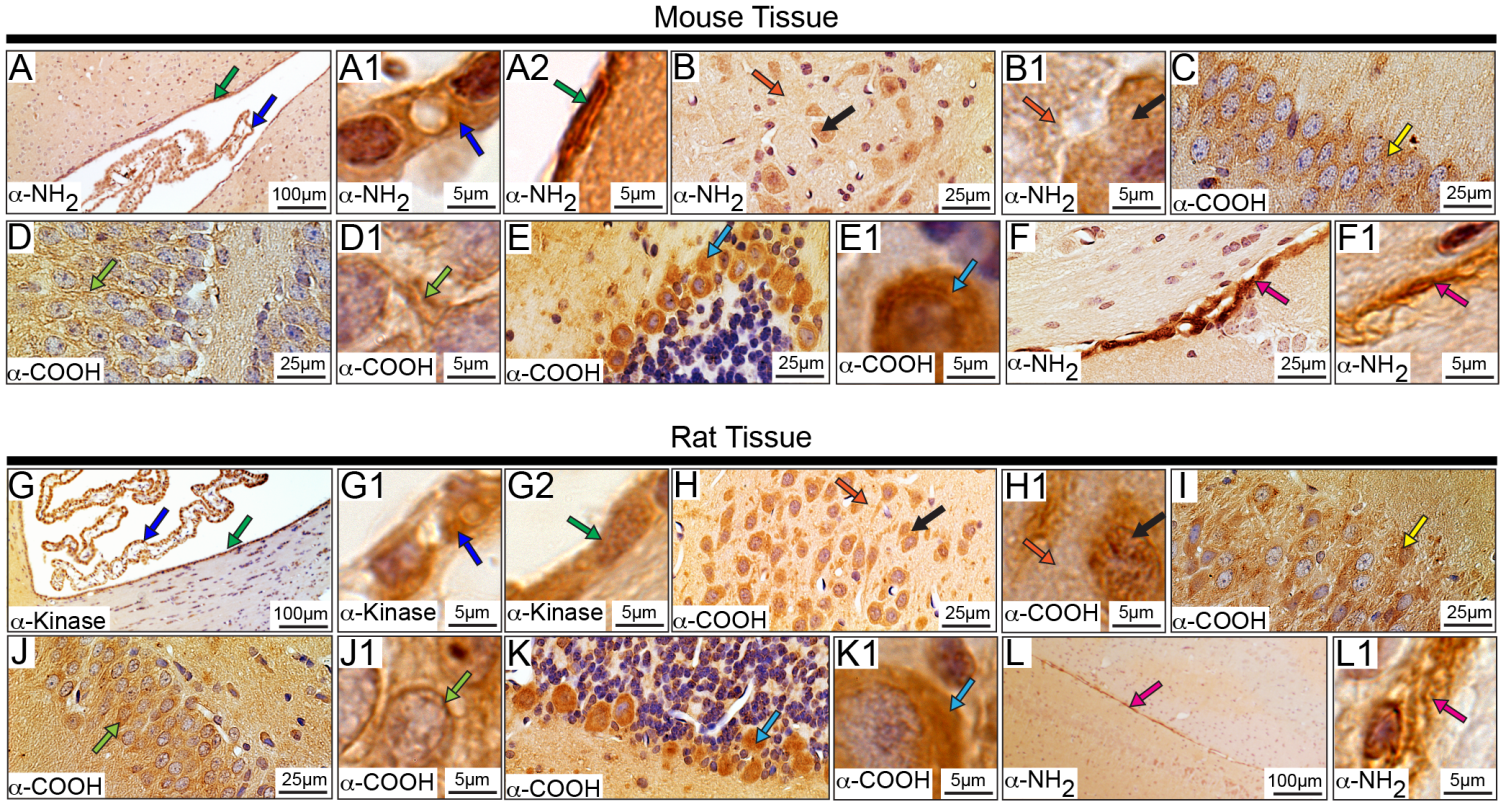
Distribution of obscurins in the rodent brain. Obscurins localize to the arachnoid (dark blue arrows) and pia mater (dark green arrows) in both mouse (A-A2) and rat (G-G2) tissue. In addition, obscurins are present in the cell bodies of neurons (black arrow) and the neuropil (orange arrows) of the mouse (B-B1) and rat (H-H1) brain. In the hippocampus, obscurins concentrate in the cytoplasm (yellow arrows) of pyramidal cells of the Cornu Ammonis (CA) both in mouse (C) and rat (I). Additionally, obscurins reside in the cytoplasm (light green arrow) of granule cells of the Dentate Gyrus (DG) of both mouse (D-D1) and rat (J-J1) tissue. Also, in the cerebellum, obscurins are present in the Purkinje cells (light blue arrows) in both mouse (E-E1) and rat (K-K1). Interestingly, fissures within the mouse (F-F1) and rat (L-L1) brain are only labeled with α-NH_2_ antibody (pink arrows). Images are shown at multiple magnifications to highlight the various immunopositive structures. Scale bars are included in each panel for reference.

Prior to the identification of the *OBSCN* gene, two partial obscurin transcripts encoding repetitive immunoglobulin domains were identified in a human brain cDNA library [22], [23], [24], [25]; one includes obscurin Ig domains 34–44 (NCBI accession number: AB046776) and the other spans from Ig domain 65 through the COOH-terminal end of obscurin-B (NCBI accession number: AB046859; [2], [22], [23], [24], [25]). Following these initial studies, however, the expression of obscurins in brain was not further investigated. Using immunoblot analysis (Figs. 2–7), we demonstrated the presence of large and small obscurin isoforms in the murine brain. We used immunohistochemical methods to examine their subcellular distribution, too (Fig. 9 and Table S2).

Three membrane layers surround and protect the brain: the dura mater, the arachnoid mater and the pia mater. The dura mater is not retained in our paraffin sections. However, obscurins are detected in both the arachnoid mater and pia mater with all four antibodies (Fig. 9 A–A2 and G–G2, dark blue and dark green arrows, respectively). Obscurins are also present in “fissures”, grooves within the brain that are aligned with the pia mater (Fig. 9 F–F1 and L–L1, pink arrows).

In the brain, obscurins localize in the cytoplasm of neurons and in the neuropil (Fig. 9 B–B1 and H–H1, black and orange arrows, respectively); an area composed of non-myelinated axons, dendrites, and glial cells. Moreover, in the hippocampus obscurins reside in the cytoplasm of granule cells present in the Dentate Gyrus (DG), as well as in the cytoplasm of pyramidal cells present in the Cornu Ammonis (CA), as observed with the α-NH_2_, α-COOH, and α-Kinase antibodies, but not with the α-ABD antibody (Fig. 9 C–D1 and I–J1, light green and yellow arrows, respectively). Interestingly though, obscurins carrying the α-ABD epitope are detected in the nuclei of both granule and pyramidal cells (data not shown). Lastly, in the cerebellum, obscurins are observed in Purkinje cells (Fig. 9 E–E1 and K–K1, light blue arrows), consistent with previous observations indicating the presence of obscurin transcripts there [22], [23], [24], [25].

### Subcellular Distribution of Obscurins in the Skin

**Figure 10:**
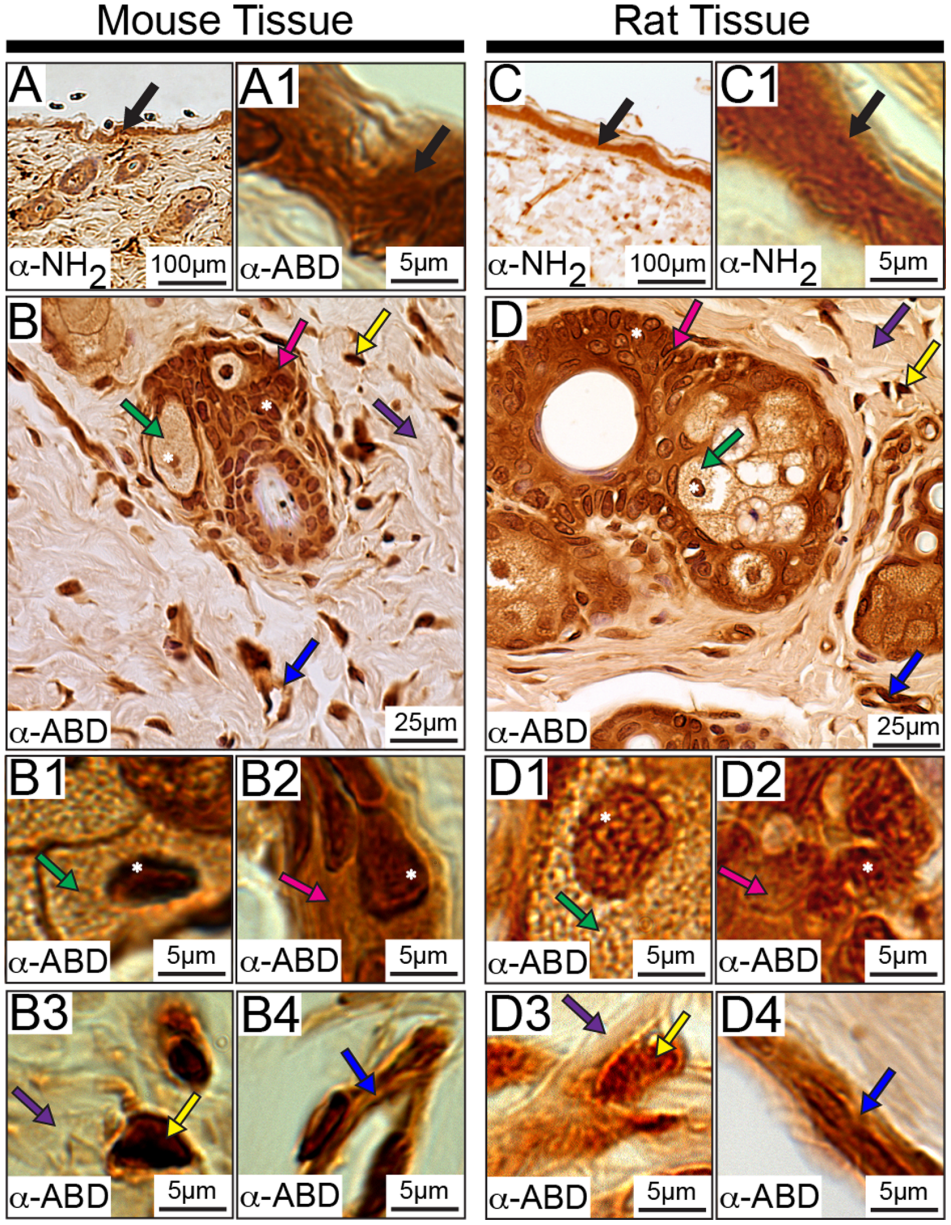
Distribution of obscurins in mouse and rat skin. Obscurins localize to the epidermis (black arrows) of mouse (A-A1) and rat (C-C1) skin. They are also found in the cytoplasm (pink arrows) and nuclei (white asterisks) of epithelial cells composing the root sheath of the hair follicle as well as the cytoplasm (green arrows) and nuclei (white asterisks) of the cells within the sebaceous glands in both mouse (B-B2) and rat (D-D2) tissue. In addition, obscurins are present in the connective tissue (purple arrows) and cells within the connective tissue (yellow arrows) in mouse (B and B3) and rat (D and D3) skin. Similar to the vasculature of striated muscles, obscurins reside within the vasculature of mouse and rat skin (B and B4, and D and D4, respectively; dark blue arrows). Images are shown at multiple magnifications to highlight the various immunopositive structures. Scale bars are included in each panel for reference.

Recent studies from our lab have demonstrated the presence of obscurins in epithelial cells derived from several human organs, including skin; within these cells, obscurins may play a key role in regulating cell survival and apoptosis [11]. Consistent with this, we observed that obscurins are present within distinct layers of mouse and rat skin, where they preferentially localize to epithelial cells (Fig. 10 and Table S2). Specifically, all four antibodies detected the presence of obscurins in the epidermis, the outermost layer of the skin, which is composed of keratinized stratified squamous epithelial cells (Fig. 10 A–A1 and C–C1, black arrows). In addition, obscurins exhibit nuclear and cytoplasmic distributions in the cuboidal epithelial cells of the root sheath and in the glandular epithelial cells of the sebaceous glands, both surrounding the hair follicle (Fig. 10 B–B2 and D–D2, pink and green arrows, respectively; nuclei are denoted by a white asterisk).

Obscurins were also detected in the fibrous component of the connective tissue (Fig. 10 B and B3, and D and D3, purple arrows), and within the cells residing in the connective tissue (Fig. 10 B and B3, and D and D3, yellow arrows). Although the precise identity of these cells is unknown, it is likely that they are fibroblasts, mast cells, or macrophages [26], [27]. Lastly, obscurins were detected in the vascular endothelial cells (VECs) present throughout the skin (Fig. 10 B and B4, and D and D4, dark blue arrows).

### Subcellular Distribution of Obscurins in the Kidney

**Figure 11:**
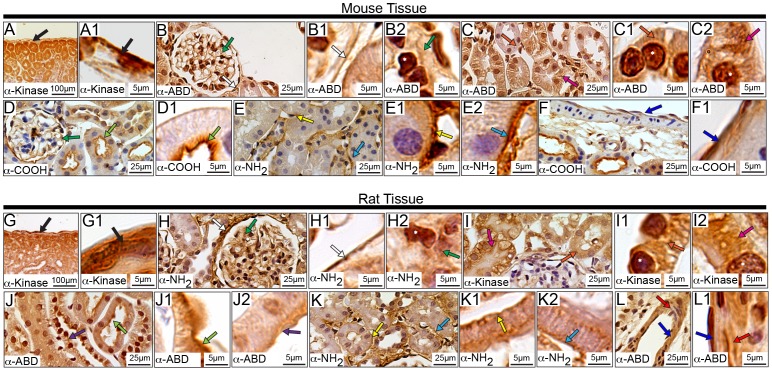
Distribution of obscurins in murine kidney. In agreement with the other tissues and organs examined, obscurins localize to the outer capsule surrounding the kidney (black arrows) in both mouse (A-A1) and rat (G-G1). Moreover, in the mouse (B and B2) and rat (H and H2) glomerulus, obscurins localize to the cytoplasm (dark green arrows) and nuclei (white asterisks) of endothelial cells. They are also found within the epithelial cells of Bowman’s capsule (B-B1 and H-H1, mouse and rat tissues, respectively; white arrows). Obscurins are also found in the cytoplasm and nuclei (white asterisks) of epithelial cells making up the proximal (pink arrows) and distal (orange arrows) tubules in both mouse (C-C2) and rat (I-I2). Notably, they are present in both the apical (light green arrows) and basolateral (yellow arrow) surfaces of the epithelial cells within distal tubules of mouse (D-E1) and rat (J-K1) tissues. Similarly, they are expressed at the basolateral surface of mouse and rat proximal tubule epithelial cells (E and E2, and K and K2, respectively; light blue arrow), with some accumulation at the apical surface in rat tissues only (J and J2, purple arrows). Obscurins also localize to the vasculature of mouse (F-F1) and rat (L-L1) kidney within both VECs (dark blue arrows) and VSMCs (red arrows). Images are shown at multiple magnifications to highlight the various immunopositive structures. Scale bars are included in each panel for reference.

Obscurins are expressed within various subsections of the kidney (Fig. 11 and Table S2), including the outer capsule composed primarily of connective tissue (Fig. 11 A–A1 and G–G1, black arrow). They are also found within the specialized types of endothelial and epithelial cells throughout the kidney. Specifically, obscurins are present in the nucleus and the cytoplasm of the endothelial cells that make up the glomerulus (Fig. 11 B, B2, D, H and H2, white asterisks and dark green arrows, respectively) and the epithelial cells within Bowman’s capsule, surrounding the glomerulus (Fig. 11 B–B1 and H–H1, white arrows). Obscurins also reside within the nuclei and cytoplasm of the cells that comprise the proximal tubule (Fig. 11 C and C2, and I and I2, white asterisks and pink arrows, respectively). Notably, obscurins possessing the α-NH_2_ epitope are enriched at the basolateral surface of epithelial cells within the proximal tubule (Fig. 11 E and E2, and K and K2, light blue arrows). As the Na^+^/K^+^ ATPase is also located at the basolateral surface of epithelial cells within the proximal tubules, it is possible that obscurins may be involved in the active transport of sodium out of the cell. This is consistent with recent findings from our group indicating that in striated muscles, obscurins associate with the extracellular region of the β1 subunit of the Na^+^/K^+^ ATPase [10]. Lastly, obscurins are found at the apical brush border surface of proximal tubule endothelial cells (Fig. 11 D–D1 and J–J1, light green arrows).

Similar to their localization in proximal tubules, obscurins were detected within the nuclei and cytoplasm of the epithelial cells that compose the distal tubules (Fig. 11 C–C1 and I–I1, white asterisks and orange arrows, respectively). Interestingly, obscurins possessing the α-NH_2_ epitope preferentially localize to the basolateral surface of distal tubule epithelial cells (Fig. 11 E–E1 and K–K1, yellow arrows), while obscurins expressing the α-ABD and α-COOH epitopes selectively accumulate at the apical surface of rat cells (Fig. 11 J and J2, purple arrows). Moreover, similarly to their localization to the vasculature of other tissues, obscurins are also present within the vasculature found throughout the kidney, in the cytoplasm of vascular smooth muscle cells (VSMCs) and VECs (Fig. 11 F–F1 and L–L1, red and dark blue arrows, respectively).

### Subcellular Distribution of Obscurins in the Liver

**Figure 12:**
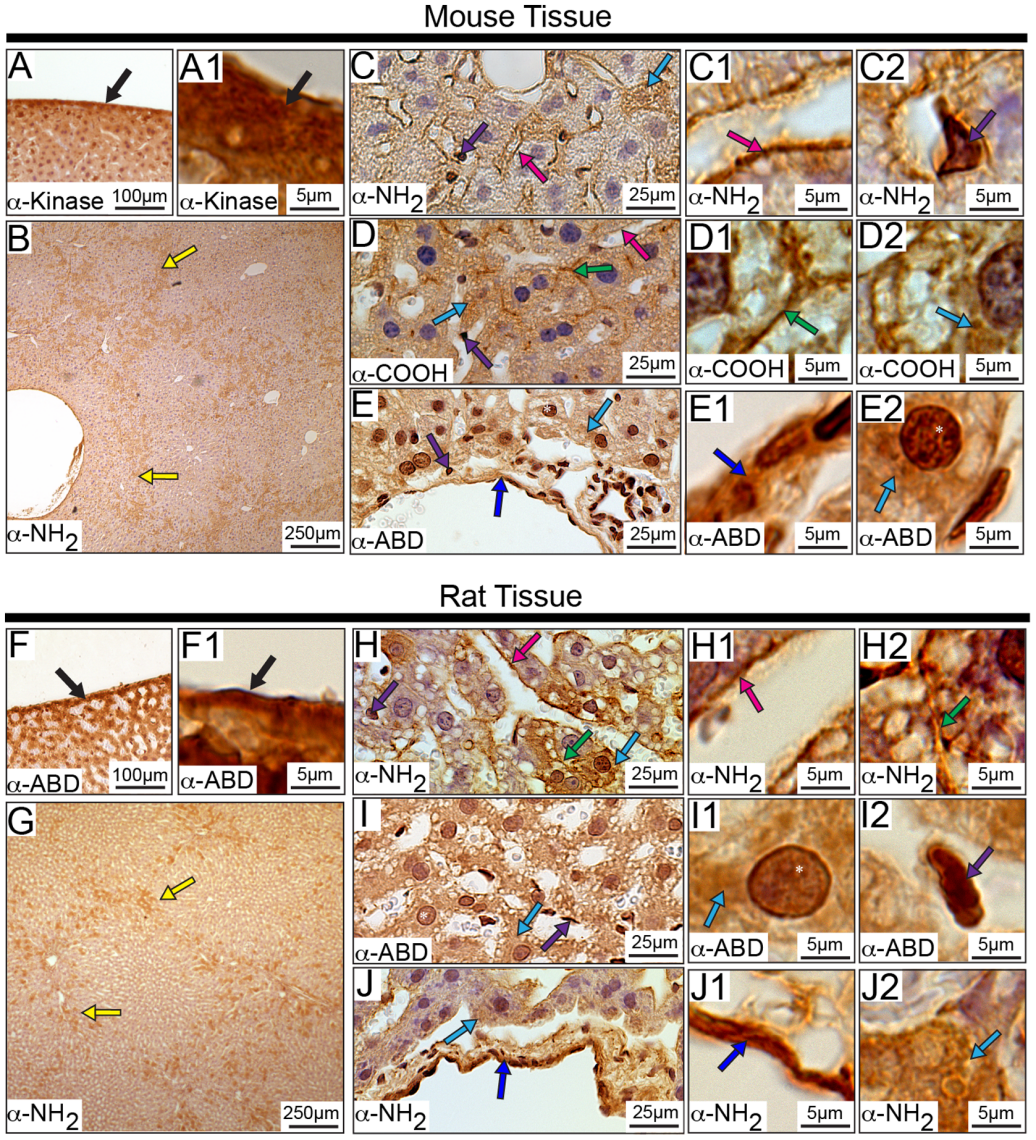
Localization of obscurins in the rodent liver. Obscurins localize to the outer surface of the liver, namely Gilsson’s capsule (black arrows) in both mouse (A-A1) and rat (F-F1) tissues. Interestingly, only epitopes at the NH_2_-terminus of obscurins are found within the connective tissue (yellow arrows) in both mouse (B) and rat (G). Obscurins are also found lining the sinusoids (C-C1, D, and H-H1, mouse and rat tissues, respectively; pink arrows) as well as within the cytoplasm of Kuppfer cells (C, C2, D and E, and H, I, and I2, mouse and rat, respectively; purple arrows) and hepatocytes (C, D, D2, E, and E2 and H, I-I1, J, and J2, mouse and rat, respectively; light blue arrows). Moreover, they are localized to the cell-cell contacts of hepatocytes (green arrows) within both mouse (D-D1) and rat (H and H2) tissue and hepatocyte nuclei (E and E2, and I-I1, mouse and rat respectively; white asterisks). Similar to other tissues and organs, obscurins reside within the VECs (dark blue arrows) of the liver vasculature in both mouse (E-E1) and rat (J-J1). Images are shown at multiple magnifications to highlight the various immunopositive structures. Scale bars are included in each panel for reference.

Obscurins are abundantly expressed in the liver (Fig. 12 and Table S2). In particular, they localize to Gilsson’s capsule, the outer surface of the liver, consisting of connective tissue and smooth muscle (Fig. 12 A–A1 and F–F1, black arrows) as well as the cytoplasm of hepatocytes (Fig. 12 C, D, D2, E, E2, H, I, I1, J, and J2, light blue arrows), as observed with all four antibodies. Interestingly, each cell type in the liver seems to express a discrete complement of obscurin isoforms, further suggesting that specific obscurins may have functionally adapted to play specialized roles in particular cells. Obscurins carrying the α-NH_2_ epitopes reside in the connective tissue separating the quadrants of the liver (Fig. 12 B and G, yellow arrows), obscurins possessing the α-ABD and α-Kinase epitopes accumulate in the nuclei of hepatocytes (Fig. 12 E, E2, and I–I1, white asterisks), while obscurins carrying the α-NH_2_ and α-COOH epitopes localize to cell-cell contacts between hepatocytes (Fig. 12 D–D1, H, and H2, green arrows). Moreover, obscurins possessing epitopes for the α-NH_2_, α-COOH, and α-Kinase antibodies were detected at the lining of sinusoids, which are primarily composed of endothelial cells (Fig. 12 C, C1, D, H, and H1, pink arrows). Obscurins are also expressed in the cytoplasm of Kupffer cells, which are specialized macrophages, as observed with all four antibodies (Fig. 12 C, C2, E, H, I, and I2, purple arrows).

### Subcellular Distribution of Obscurins in the Spleen

**Figure 13:**
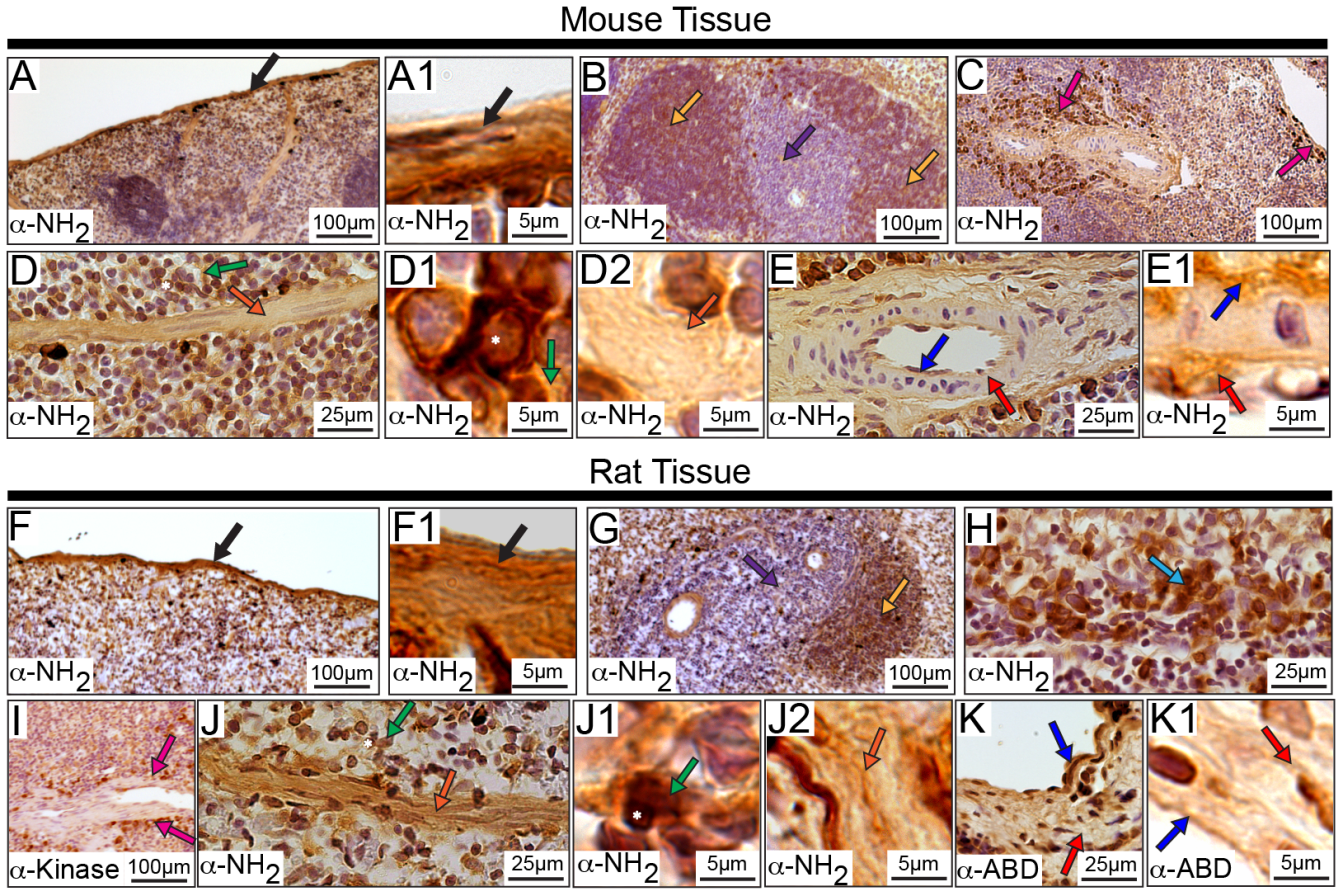
Distribution of obscurins in the rodent spleen. Obscurins are present in the outer capsule (black arrows) of the mouse (A-A1) and rat (F-F1) spleen. In addition, they are expressed in the lymphocytes of B-cell follicles of the white pulp (yellow arrows) in both mouse (B) and rat (G). However, obscurins are absent from the T-cell area (purple arrows) surrounding the central arteriole of either mouse (B) or rat (G) spleen. Only those obscurins carrying NH_2_-terminal epitopes are found within the perivascular region (pink arrows) of both mouse (C) and rat (I) spleen. Unique to rat spleen (H), the cells within the marginal zone surrounding the white pulp are immunopositive for obscurins carrying the α-NH_2_ and α-kinase epitopes (light blue arrows). Moreover, obscurins are detected in both the cytoplasm (green arrows) and nuclei (white asterisks) of cells residing in the red pulp of both mouse (D-D1) and rat (J-J1) spleen. Obscurins are also present within the trabeculae (orange arrows) of both mouse (D and D2) and rat (J and J2). Furthermore, we observe staining of VSMCs (red arrows) and VECs (dark blue arrows) in both mouse (E-E1) and rat (K-K1) spleen. Images are shown at multiple magnifications to highlight the various immunopositive structures. Scale bars are included in each panel for reference.

Within the murine spleen, obscurins are ubiquitously expressed (Fig. 13 and Table S2). Similar to the rodent kidney and liver, obscurins, detected with all four antibodies used, reside in the outer capsule of the spleen, which is composed of connective tissue and smooth muscle (Fig. 13 A–A1 and F–F1, black arrows). The spleen is mainly comprised of two regions: the red pulp and the white pulp. Obscurins exhibit nuclear and cytoplasmic staining in the cells that make up the red pulp (Fig. 13 D–D1 and H–H1, white asterisks and green arrows, respectively). Moreover, obscurins localize to the B-cell follicle within the white pulp (Fig. 13 B and G, yellow arrows). On the contrary, obscurins are absent from the T-cell region (Fig. 13 B and G, purple arrows), suggesting that they may play important roles in B-cell specific functions, such as antibody production.

Select obscurins localize to the cells of the perivascular region in both mouse and rat, and of the marginal zone surrounding the white pulp in rat (Fig. 13 C and I, pink arrows, and H, light blue arrow, respectively). While the identity of these cells, which appear to express high levels of obscurins, is unknown, based on their localization it is likely that they are macrophages or other phagocytes [28]. This interpretation is consistent with the presence of obscurins in the Kupffer macrophages of the liver.Obscurins were also detected within the vasculature of the spleen. In particular, they reside within the trabeculae, primarily composed of vascular smooth muscle (Fig. 13 D and J, orange arrows), as well as the large and small vessels composed of VSMCs and VECs (Fig. 13 E–E1 and K–K1, red and dark blue arrows, respectively).

### Subcellular Distribution of Obscurins in the Lung

**Figure 14:**
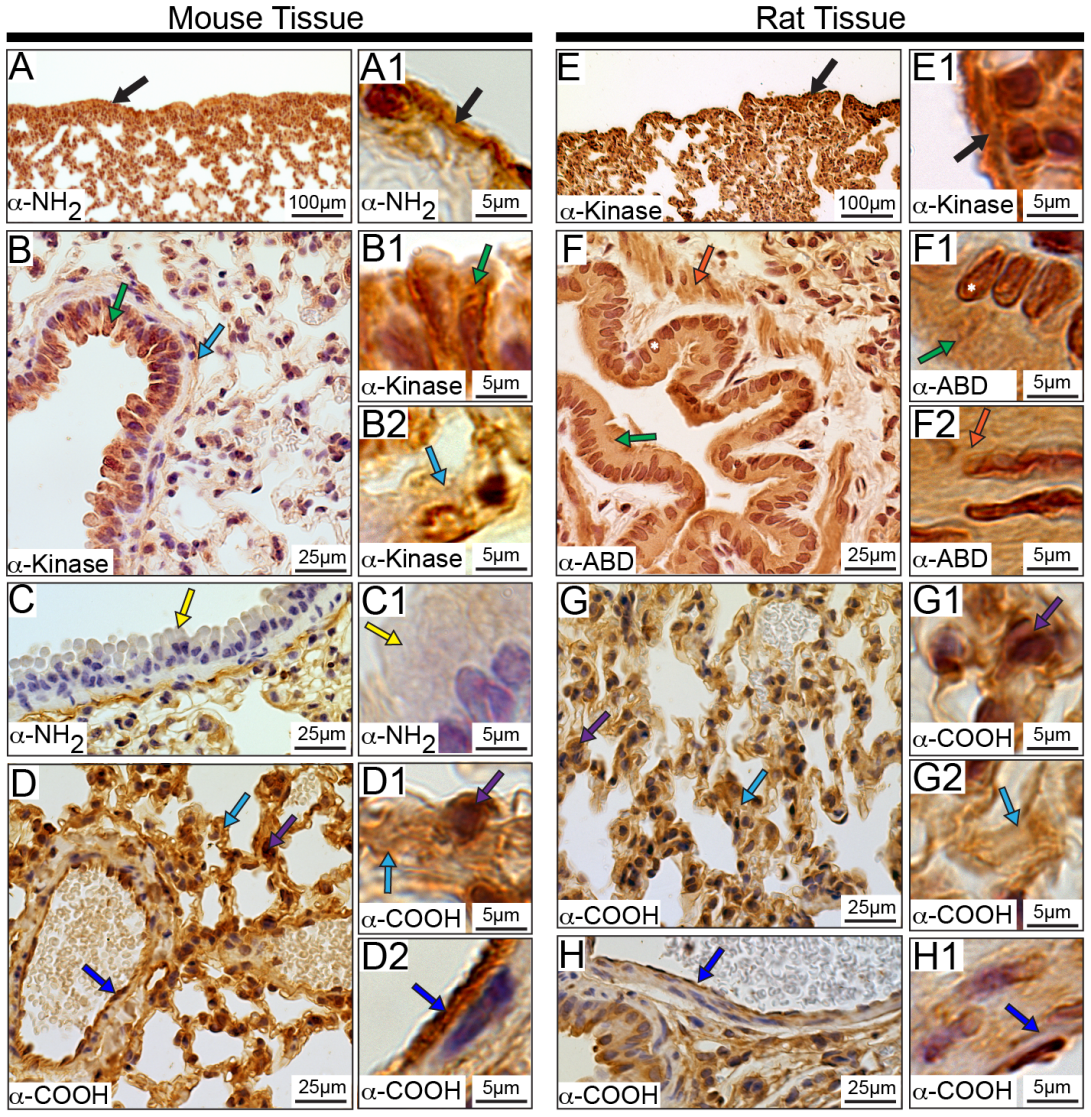
Distribution of obscurins in rodent lung. Obscurins localize to the mesothelial cells making up the pleura, which surrounds the lung (A-A1 and E-E1, mouse and rat, respectively; black arrows). Within the bronchioles, particular obscurins are found in the cytoplasm of Clara cells (green arrows) of both mouse (B-B1) and rat (F-F1) lung. Interestingly, obscurins carrying the NH_2_-terminal epitopes are absent from the cytoplasm of Clara cells in mouse tissue (C-C1, yellow arrows). Obscurins are also found in the fibrous component of the connective tissue (B, B2, D-D1, G, and G2, light blue arrows) as well as in cells within the connective tissue (purple arrow) in both mouse (D-D1) and rat (G-G1) lung. Similar to observations in other tissues, obscurins localize to VECs (dark blue arrows) in the mouse (D and D2) and rat (H-H1) lung. Smooth muscle within surrounding the bronchioles contains obscurins only in rat tissue (F and F2, orange arrows). Images are shown at multiple magnifications to highlight the various immunopositive structures. Scale bars are included in each panel for reference.

We observed expression of obscurins in a number of the diverse cell types that make up the lungs (Fig. 14 and Table S2). Obscurins reside within the pleura, which consists of mesothelial cells and connective tissue (Fig. 14 A–A1 and E–E1, black arrows). The cytoplasm of Clara cells are stained by most antibodies (Fig. 14 B–B1 and F–F1, green arrows), however, immunoreactivity to the α-NH_2_ epitope is completely absent in mouse cells (Fig. 14 C–C1, yellow arrows). Notably, select obscurins containing the α-ABD epitope are also detected in the nuclei of Clara cells in rat, but not mouse, lung (Fig. 14 F–F1, white asterisks).

Consistent with their expression in the connective tissue of other organs, obscurins are also present in the fibrous component of the connective tissue of the lungs, as shown with all four antibodies (Fig. 14 B, B2, D–D1, G, and G2, light blue arrows), in addition to the cells found throughout the connective tissue, likely macrophages or type II pneumocytes (Fig. 14 D–D1 and G–G1, purple arrows) [26], [27]. Similarly, obscurins are abundantly expressed in the cytoplasm of VECs lining the vasculature found throughout the lung, (Fig. 14 D–D2 and H-H1, dark blue arrows). However, in lung tissue, expression of obscurins in smooth muscle seems limited to rat tissue α-ABD and α-NH_2_ epitopes, and includes the smooth muscle surrounding the bronchioles (Fig. 14 F and F2, orange arrows).

### Obscurins Comprise a Complex Family of Proteins

To date, most studies on obscurins have focused on the role of the giant isoforms in striated muscles [1], [3], [4], [7], [8], [9], [12], [16], [17], [19], [20], [21], [29], [30], [31], [32], [33], [34], [35], [36], [37], [38], [39], [40], [41], [42], [43], [44], [45], [46], [47], [48], [49], [50], [51], [52], [53]. Herein, we have begun to examine the expression profile and subcellular distribution of small (∼50–260 kDa), intermediate (∼260–600 kDa), and giant (>600 kDa) obscurins in diverse mouse and rat tissues and organs. Our studies demonstrate for the first time that obscurins comprise a large family of proteins consisting of over twenty isoforms that range in size between ∼50–900 kDa and are expressed in muscle and non-muscle tissues. Based on our western blot analysis, some obscurin isoforms are ubiquitously expressed, likely serving similar functions, while others are expressed in a tissue-specific manner, possibly having unique roles. Our immunohistochemical analysis further revealed that obscurins share similar subcellular distributions across different tissues and organs, residing in the nucleus, the cytosol, and/or the cell membrane. However, the limited resolution of our immunohistochemical analysis does not allow us to specify the subcellular organelle or membrane compartment in which they accumulate in each cell type. In addition, obscurins are expressed in structures shared by many organs - the outer capsule, vasculature, and connective tissue - suggesting functional commonality.

Our studies are the tip of the iceberg in comprehending the obscurin subfamily. A detailed molecular characterization of the *OBSCN* transcripts is required to understand their diverse expression profile. Moreover, dissecting the precise role that each obscurin isoform plays is an ambitious task that will require a combination of molecular, cellular, and biochemical approaches alongside the generation of the appropriate animal models. Future work is therefore warranted to decipher the exact role of individual obscurins in diverse tissues and organs during development and adulthood, in normalcy and disease. Given the immense complexity of the multifaceted obscurin subfamily, it is imperative that we tailor our questions, hypotheses, and methodologies in such ways that will allow the methodical and comprehensive characterization of each obscurin’s molecular identity, properties, and regulation with respect to its tissue and organ expression. Although the studies presented herein are limiting in their description of individual obscurins, they provide a platform for the initiation of systematic work focusing on the molecular and functional characterization of the many obscurin isoforms expressed in various tissues and organs.

Taken together, our studies show for the first time that obscurins are abundantly expressed in several tissues and organs throughout the body. Their functions remain to be determined; however, given their structural, scaffolding, and signaling roles in striated muscles and mammary epithelial cells, we can speculate that they are essential to maintaining cellular organization and contributing to signal transduction. While extensive work is still needed to molecularly and functionally characterize the small, intermediate, and giant obscurins in muscle and non-muscle tissues, obscurins are no longer “obscure,” and comprise an exciting and diverse new family for cell biologists to explore.

## Materials and Methods

### Database Search

Following a thorough search of three prominent databases - NCBI (http://www.ncbi.nlm.nih.gov/), Ensembl (http://www.ensembl.org), and Vega (http://vega.sanger.ac.uk/index.html) - we identified several mammalian obscurin isoforms. To classify the product of a transcript as a complete isoform the following guidelines were set: 1. the transcript contained both a start and stop codon, 2. the transcript contained at least partial 5′ and/or 3′ UTRs, and 3. the transcript encoded a known region of obscurin. Human and mouse isoforms are included in Table 1, while other mammalian obscurins are listed in Table S1. Human and mouse obscurins A and B, human obscurin-970, human obscurin-20, cow obscurin-90, and baboon obscurin-70 were identified in all three databases (Table 1 and Table S1; for simplicity, only the NCBI accession number is provided). The remaining fourteen isoforms were found in the Ensembl and Vega databases (Table 1 and Table S1; for simplicity, only the Ensembl accession number is noted).

### Tissue Collection

Female C57BL6 mice and Sprague-Dawley rats were perfused with either phosphate buffered saline with protease inhibitors (Roche Applied Science, Indianapolis, IN) or 4% paraformaldehyde for preparation of protein lysates or tissue sections analyzed by western blotting or immunohistochemistry, respectively. Following dissection, the indicated tissues and organs (i.e. heart, tibialis anterior, quadriceps, soleus, and diaphragm muscles, as well as brain, skin, kidney, liver, spleen, and lung) were either snap-frozen for generation of protein lysates, or soaked in formalin for 14 hours followed by storage in 100% ethanol for preparation of sections.

All animals were housed and treated in accordance with IACUC guidelines and protocols. Specifically, they were euthanized under isofluorane during the perfusion protocol. All efforts were made to minimize suffering and maintain comfort. This study was carried out via the instructions from the Guide for the Care and Use of Laboratory Animals of the National Institutes of Health. The protocol was reviewed and approved by the committee on Ethics of Animal Care and Experimentation at the University of Maryland, Baltimore (IACUC protocol number: 0111006).

### Western Blotting

Lysates from each tissue were prepared as previously described [3]. Briefly, tissues were homogenized in 10 mM NaPO_4_, pH 7.2, 2 mM EDTA, 10 mM NaN_3_, 120 mM NaCl, and 1% NP-40 in the presence of protease inhibitors (Roche Applied Science), incubated on ice for 2 hours with occasional mixing, and centrifuged at 14,000×g for 30 minutes at 4°C. The Bradford assay (BioRad, Hercules, CA) was used to measure lysate concentration, and 70 µg of protein from each tissue or organ were separated by SDS-PAGE using the precast Invitrogen system (Life Technologies, Carlsbad, CA). For the large and intermediate isoforms (>260 kDa) 3–8% tris-acetate gels were used, while for the small isoforms (<260 kDa) 4–12% bis-tris gels were used. Gels were transferred to nitrocellulose (15 V, 16 hours, 4°C) and probed with antibodies to different regions of obscurins. Equal loading was evaluated by blotting with a GAPDH antibody (1∶5000; Sigma) and confirmed with Ponceau staining, and experiments were replicated at least 3 times. The molecular weight of each immuno-reactive band was calculated using standard methods as previously described [54]. For ease of presentation, we have segmented each immunoblot into three parts, shown in figures 2 (including the very top of the gel through ∼600 kDa), 4 (containing proteins between ∼600 kDa –260 kDa), and 6 (including proteins between ∼260 kDa –40 kDa).

Antibodies to obscurins included: a mouse α-NH_2_ antibody, directed against the first Ig domain (Ig1, residues 1–100, accession number NM_001098623) (1.5 µg/ml, [3]), a rabbit α-COOH antibody, recognizing the two Ig domains just after the RhoGEF/PH module (Ig65/Ig66, residues 6014–6200, accession number NM_052843) (300 ng/ml, [8]), a rabbit α-ABD antibody, produced against the Ankyrin-Binding Domain (ABD) within the extreme COOH-terminus of obscurin-A variants (400 ng/ml, residues 6311–6431, accession number NM_052843), and a rabbit α-Kinase antibody, recognizing the FNIII domain flanking the second kinase domain at the extreme COOH-terminus of obscurin-B variants (200 ng/ml, residues 7554–7648, accession number NM_001098623 [10]). The rabbit α-ABD antibody was generated using a rabbit GST-ABD fusion protein, encompassing the second ankyrin-binding domain (amino acids 6312–6432, accession number: NM_052843; [8]). Antiserum was affinity purified sequentially using cyanogen bromide-coupled columns specific for GST and GST-ABD. The specificity of the immunoreactive bands observed with each one of the aforementioned obscurin antibodies was verified in immunodepletion experiments, as described in [8].

### Immunohistochemistry

Murine heart, tibialis anterior, brain, skin, kidney, liver, spleen, and lung were prepared for immunohistochemical analysis. First, each tissue was paraffin-embedded and sectioned at 5 µm thickness. The sections were then sequentially deparaffinized with two washes in each of the following solutions: xylene, absolute ethanol, 95% ethanol, and 70% ethanol. Next, sections were prepared for staining with one of the four obscurin antibodies α-NH_2_, α-COOH, α-ABD, and α-Kinase. For staining with the α-COOH and α-kinase antibodies, tissue sections were digested with Target Retrieval Solution (Dako Inc., Glostrup, Denmark) for 20 minutes, washed several times with PBS, and incubated in 100% methanol with 0.3% H_2_O_2_ for 30 minutes, to exhaust the endogenous peroxidase activity. For staining with the α-NH_2_ and α-ABD antibodies, tissue sections were incubated for 30 minutes in 100% methanol with 0.3% H_2_O_2_, and then incubated with ficin protease (1∶50) at 37°C for 30 minutes. The sections were then blocked in 10% horse serum for 20 minutes at RT, followed by primary antibody incubation (10 ng/µl) for 1 hour at room temperature, and biotinylated secondary α-mouse or α-rabbit antibody for 30 minutes. After extensive washing with PBS, the sections were incubated with ABC reagent (Vector Laboratories Inc., Bulingame, CA), followed by incubation with 3,3-diaminobenzidine (DAB Peroxidase Substrate Kit, Vector Labroatories Inc.) for 3 minutes, and counterstained with Mayer’s Hematoxylin for 2 minutes. The sections were dehydrated with sequential washes of 95% ethanol, absolute ethanol, and xylene. Dehydrated sections were mounted on glass slides and analyzed using an inverted fluorescent microscope (Olympus IX51) with 4× (250 µm scale bar), 10× (100 µm scale bar), 40× (25 µm scale bar), and 100× (5 µm scale bar) objectives.

### Reagents

Unless otherwise specified, all reagents were of the highest molecular grade and purchased from Sigma-Aldrich (St. Louis, MO).

## Supporting Information

Figure S1 **Expression of GAPDH in rodent tissues and organs.** Western blot analysis of 70 µg of protein homogenates prepared from various adult mouse (A) and rat (B) tissues were probed with antibodies specific to GAPDH. Each lane is a representative image from multiple replicates.
(TIF)

Table S1 List of mammalian obscurin isoforms.
(DOCX)

Table S2 Localization of obscurins in rodent tissues.
(DOCX)
