# Correction: Obscurins: Goliaths and Davids Take over Non-Muscle Tissues

**DOI:** 10.1371/journal.pone.0190842

**Published:** 2018-01-03

**Authors:** Maegen A. Ackermann, Marey Shriver, Nicole A. Perry, Li-Yen R. Hu, Aikaterini Kontrogianni-Konstantopoulos

There is an error in Fig 6B. The “Quadricep” lane for the α-Kinase obscurin blot is incorrect. Please see the corrected Fig 6 here.

**Fig 6:**
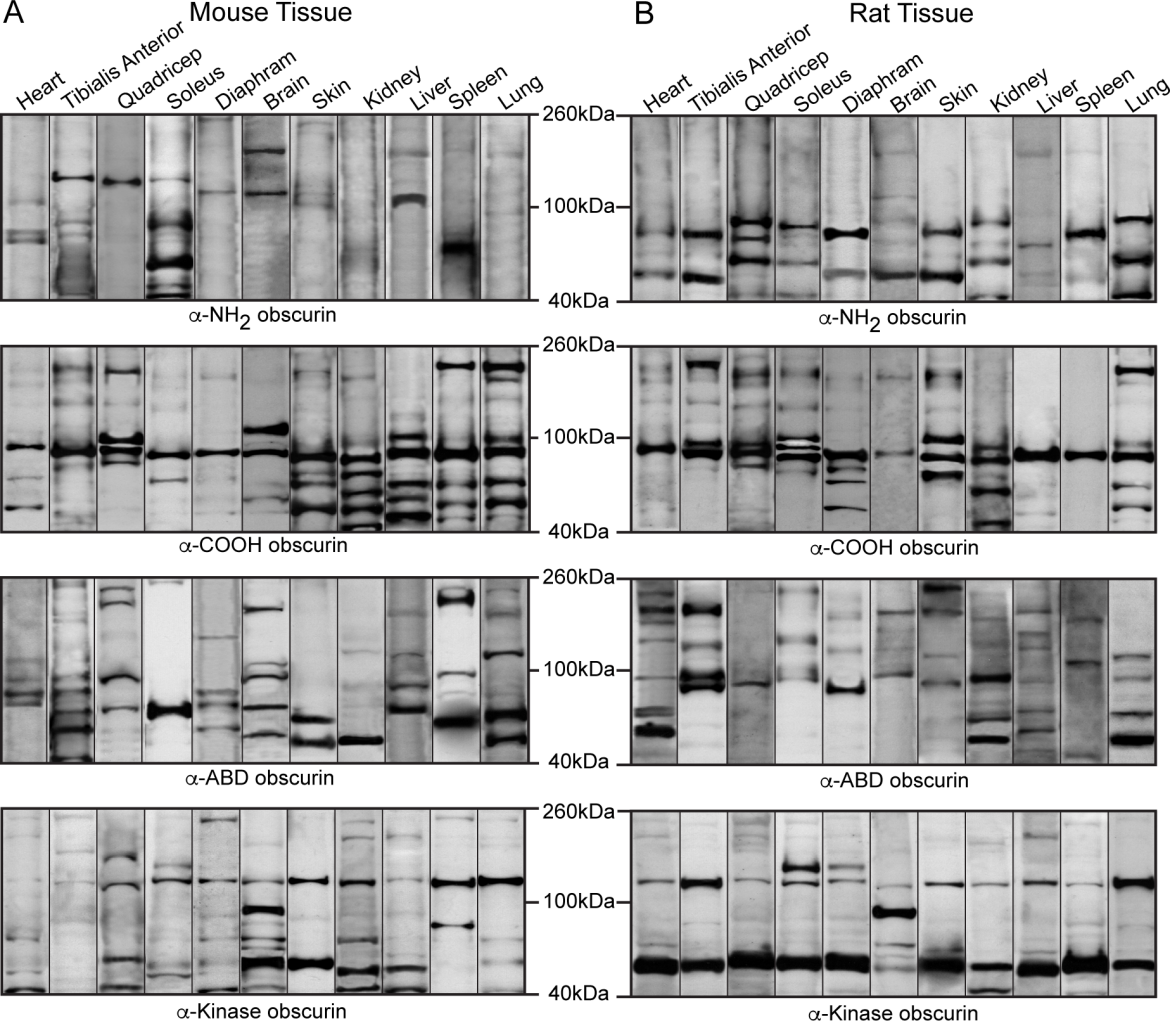
Expression of small obscurins in rodent tissues and organs. Western blot analysis of 70 μg of protein homogenates prepared from various adult mouse (A) and rat (B) tissues were probed with antibodies specific to obscurins and a GAPDH loading control. The blots have been cut to show small obscurins with molecular weights of ∼40–260 kDa. A representative blot for each tissue is shown in every lane.

There is an error in Fig 7. The “Quadricep” antibodies in columns J and V are incorrect. Please see the corrected Fig 7 here.

**Fig 7:**
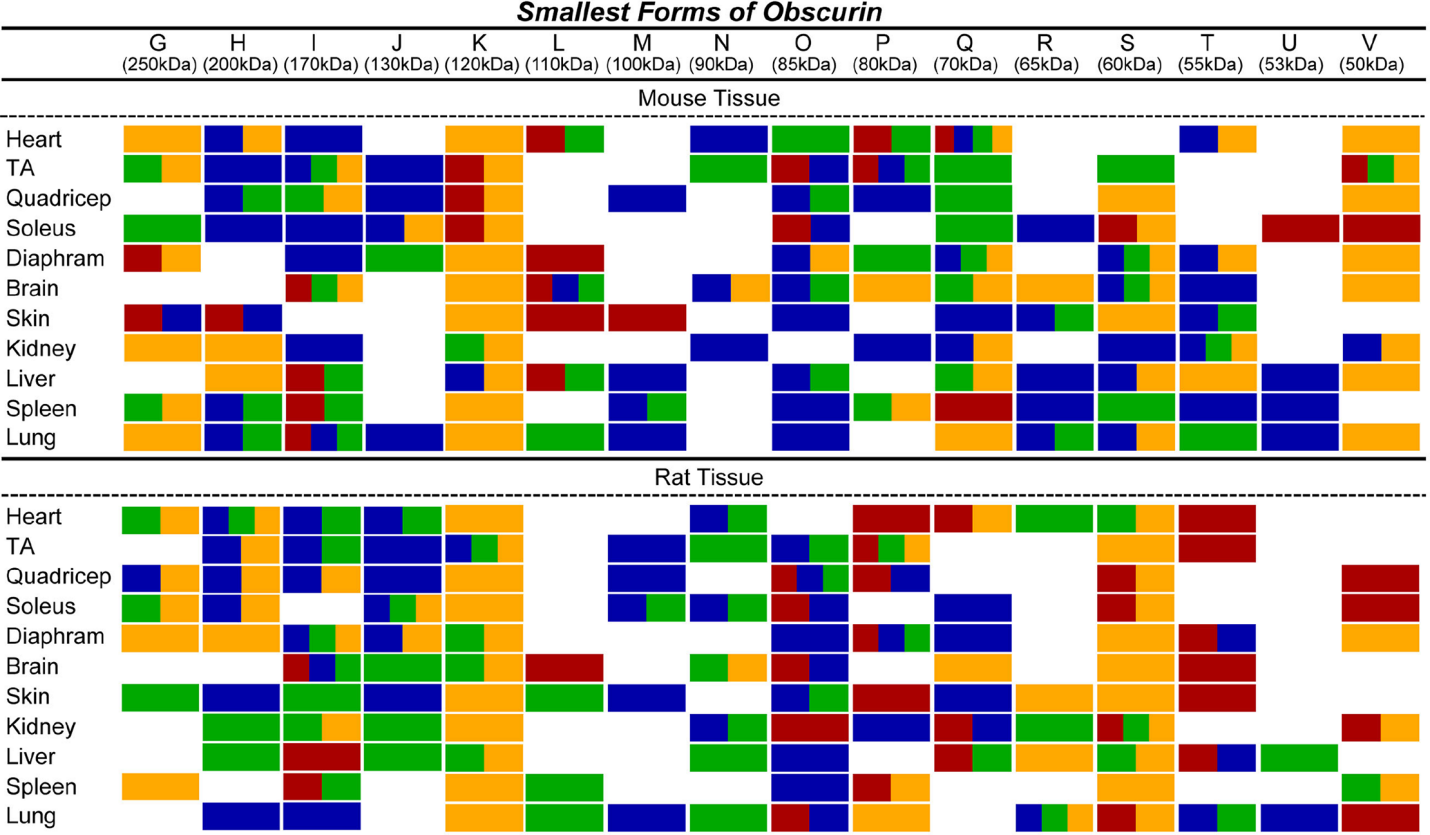
Epitopes present in small obscurins. The ability of each of the four obscurin antibodies (α-NH_2_ in red, α-COOH in blue, α-ABD in green, and α-Kinase in yellow) to recognize small obscurins (∼40–260 kDa) is depicted for each murine tissue and organ.
