# Supplementary figures and images for: Obscurins: Goliaths and Davids Take over Non-Muscle Tissues
Source: PLoS One. 2014 Feb 6;9(2):e88162. doi: 10.1371/journal.pone.0088162 (PMC3916441; doi:10.1371/journal.pone.0088162)

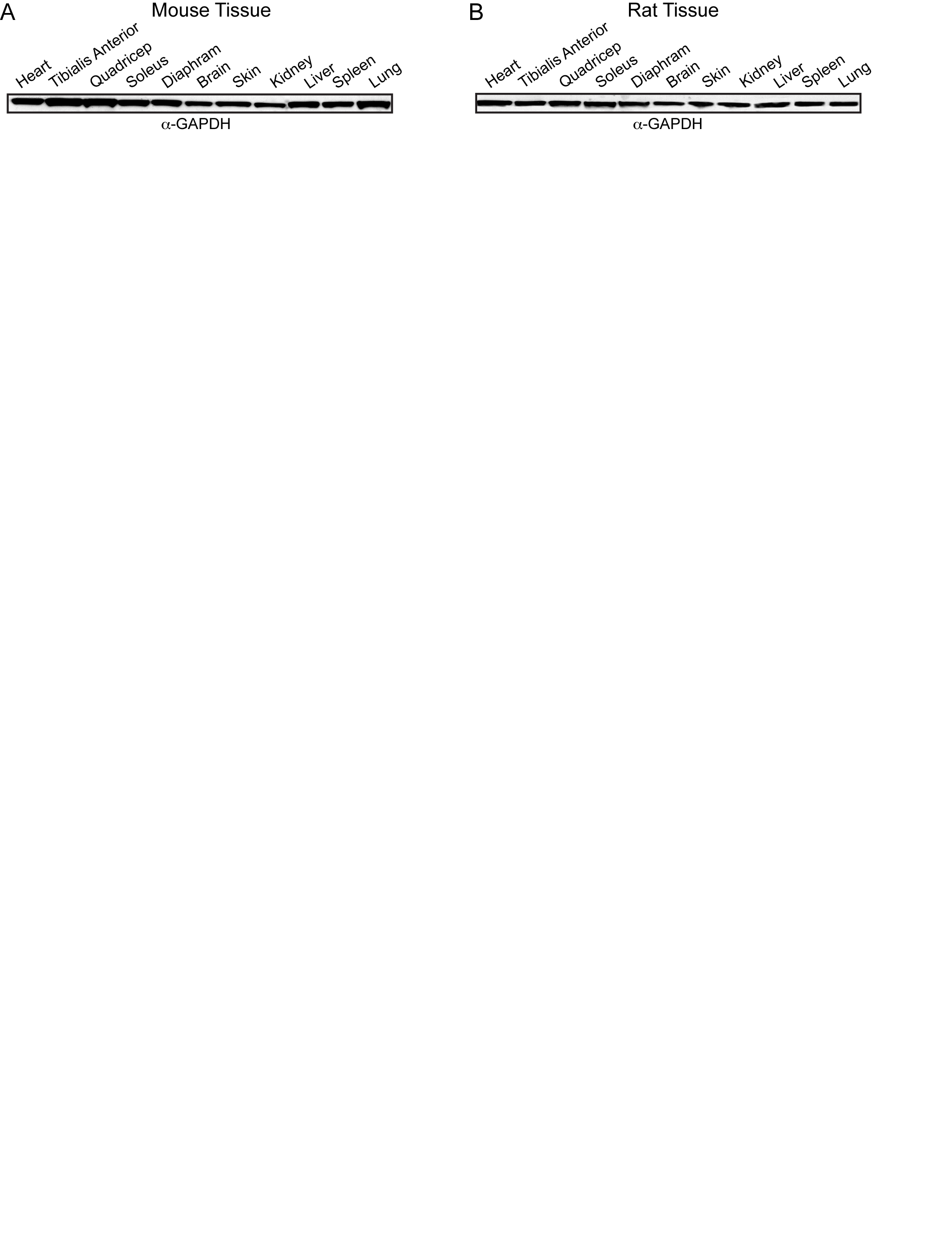

Supplement: Figure S1 — Expression of GAPDH in rodent tissues and organs. Western blot analysis of 70 µg of protein homogenates prepared from various adult mouse (A) and rat (B) tissues were probed with antibodies specific to GAPDH. Each lane is a representative image from multiple replicates. (TIF) [file pone.0088162.s001.tif]
