# Supplementary material for: Obscurins: Goliaths and Davids Take over Non-Muscle Tissues
Source: PLoS One. 2014 Feb 6;9(2):e88162. doi: 10.1371/journal.pone.0088162 (PMC3916441; doi:10.1371/journal.pone.0088162)
Supplement: Table S1 — List of mammalian obscurin isoforms. (DOCX) [file pone.0088162.s002.docx]

Table S1: List of mammalian obscurin isoforms.

| **Notation** | **Species** | **Mol Wt (kDa)** | **Contains the epitope for:** | | | | | | | **Transcript Database (accession #)** |
| --- | --- | --- | --- | --- | --- | --- | --- | --- | --- | --- |
|  |  |  | **α-NH_2_** | **α-COOH** | | **α-ABD** | | | **α-Kinase** |  |
| ***Obscurin-A-like Isoforms*** | | | | | | | | | | |
| Obscurin-A | Opossum | 730 | + | | + | | + | - | | Ensembl (ENSMODP00000017211) |
| Obscurin90 | Cow | 90 | - | | + | | + | - | | NCBI (NM_001102196)^#^ |
| ***Obscurin-B-like Isoforms*** | | | | | | | | | | |
| Obscurin-B | Horse | 840 | + | | + | | - | + | | Ensembl (ENSECAP00000014761) |
| Obscurin-B | Dolphin | 840 | + | | + | | - | + | | Ensembl (ENSTTRP00000013567) |
| Obscurin-B | Megabat | 840 | + | | + | | - | + | | Ensembl (ENSPVAP00000008878) |
| Obscurin-B | Orangutan | 835 | + | | + | | - | + | | Ensembl (ENSPPYP00000000139) |
| Obscurin220 | Pig | 220 | - | | + | | - | + | | Ensembl (ENSSSCP00000014879) |
| Obscurin50 | Cow | 50 | - | | - | | - | + | | Ensembl (ENSBTAP00000002103) |
| ***Additional Obscurin Isoforms*** | | | | | | | | | | |
| Obscurin170 | Macaque | 170 | - | | - | | - | - | | Ensembl (ENSMMUP00000030406) |
| Obscurin80 | Macaque | 80 | - | | - | | - | - | | Ensembl (ENSMMUP00000038473) |
| Obscurin70 | Baboon | 70 | - | | - | | - | - | | NCBI (NM_001168742) |
| Obscruin60 | Platypus | 60 | + | | - | | - | - | | Ensembl (ENSOANP00000030131) |

# Also found in Ensembl.
