# Supplementary material for: Obscurins: Goliaths and Davids Take over Non-Muscle Tissues
Source: PLoS One. 2014 Feb 6;9(2):e88162. doi: 10.1371/journal.pone.0088162 (PMC3916441; doi:10.1371/journal.pone.0088162)
Supplement: Table S2 — Localization of obscurins in rodent tissues. (DOCX) [file pone.0088162.s003.docx]

Table S2: Localization of obscurins in rodent tissues.

| **Localization within the tissue** | | **Obscurin Antibody Labeling** | | | | | | | | |
| --- | --- | --- | --- | --- | --- | --- | --- | --- | --- | --- |
|  |  | **α-NH_2_** | | | **α-COOH** | | **α-ABD** | | **α-kinase** | |
|  | | **e** | **rat** | | **e** | **rat** | **e** | **rat** | **e** | **rat** |
| *Cardiac Muscle* | | | | | | | | | | |
| Mesothelial cells within the epicardium | | **+** | **+** | | **+** | **+** | **+** | **+** | **+** | **+** |
| Myo-  cardium | Sarcolemma | **+** | **+** | | **+** | **+** | **-** | **+** | **+** | **+** |
|  | Intercalated Disc (ICD) | **-** | **-** | | **+** | **+** | **+** | **+** | **+** | **+** |
|  | Sarcomeric striations | **+** | **+** | | **+** | **+** | **+** | **+** | **+** | **+** |
|  | Nucleus | **-** | **-** | | **-** | **-** | **+** | **+** | **+** | **-** |
| Vasculature | | **+** | **+** | | **+** | **+** | **+** | **+** | **+** | **+** |
| *Skeletal Muscle* | | | | | | | | | | |
| Myo-  fiber | Sarcolemma | **+** | | **+** | **+** | **+** | **-** | **+** | **+** | **+** |
|  | Sarcomeric Striations | **+** | | **+** | **+** | **+** | **+** | **+** | **+** | **+** |
|  | Nucleus | **-** | | **-** | **-** | **-** | **+** | **-** | **-** | **-** |
| Vasculature | | **+** | | **+** | **+** | **+** | **+** | **+** | **+** | **+** |
| *Brain* | | | | | | | | | | |
| Fissure | | **+** | | **+** | **-** | **-** | **-** | **-** | **-** | **-** |
| Pia mater | | **+** | | **+** | **+** | **+** | **+** | **+** | **+** | **+** |
| Arachnoid mater | | **+** | | **+** | **+** | **+** | **+** | **+** | **+** | **+** |
| Neuron | | **+** | | **+** | **+** | **+** | **+** | **+** | **+** | **+** |
| Neuropil | | **+** | | **+** | **+** | **+** | **+** | **+** | **+** | **+** |
| Hippo-  campus | Cytoplasm of cells within the dentate gyrus | **+** | | **+** | **+** | **+** | **-** | **-** | **-** | **+** |
|  | Nucleus of cells within the dentate gyrus | **-** | | **-** | **-** | **-** | **+** | **+** | **-** | **-** |
|  | Cytoplasm of cells within the cornu annois | **+** | | **+** | **+** | **+** | **-** | **+** | **+** | **+** |
|  | Nucleus of cells within the cornu annois | **-** | | **-** | **-** | **-** | **+** | **+** | **-** | **-** |
| Purkinje cells within the cerebellum | | **+** | | **+** | **+** | **+** | **+** | **+** | **+** | **+** |
| *Skin* | | | | | | | | | | |
| Epidermis | | **+** | | **+** | **+** | **+** | **+** | **+** | **+** | **+** |
| Root sheath of the hair follicle | | **+** | | **+** | **+** | **+** | **+** | **+** | **+** | **+** |
| Seba-ceous  Gland | Cytoplasm | **+** | | **+** | **+** | **+** | **+** | **+** | **+** | **+** |
|  | Nuclei | **+** | | **-** | **+** | **+** | **+** | **+** | **+** | **+** |
| Connective tissue | | **+** | | **+** | **+** | **-** | **+** | **+** | **+** | **+** |
| Cells within the connective tissue | | **+** | | **+** | **+** | **+** | **+** | **+** | **+** | **+** |
| Vasculature | | **+** | | **+** | **-** | **+** | **+** | **+** | **+** | **+** |
| *Kidney* | | | | | | | | | | |
| Capsule | | **+** | | **+** | **-** | **+** | **-** | **+** | **+** | **+** |
| Cytoplasm of cells within the glomerulus | | **+** | | **+** | **+** | **+** | **+** | **+** | **+** | **+** |
| Nucleus of cells within the glomerulus | | **_** | | **+** | **-** | **-** | **+** | **+** | **-** | **-** |
| Proximal  Tubule | Apical Surface | **-** | | **-** | **-** | **+** | **-** | **+** | **-** | **+** |
|  | Basolateral Surface | **+** | | **+** | **-** | **-** | **-** | **-** | **-** | **-** |
|  | Cytoplasm | **+** | | **+** | **+** | **+** | **+** | **+** | **+** | **+** |
|  | Nuclei | **-** | | **-** | **-** | **+** | **+** | **+** | **+** | **+** |
| Distal  Tubule | Apical Surface | **-** | | **-** | **-** | **+** | **-** | **+** | **-** | **-** |
|  | Basolateral Surface | **+** | | **+** | **-** | **-** | **-** | **-** | **-** | **-** |
|  | Cytoplasm | **+** | | **+** | **+** | **+** | **+** | **+** | **+** | **+** |
|  | Nuclei | **-** | | **-** | **-** | **+** | **+** | **+** | **-** | **+** |
| Vascu-  lature | Smooth muscle cells | **+** | | **+** | **-** | **-** | **+** | **+** | **-** | **-** |
|  | Endothelial cells | **+** | | **+** | **+** | **+** | **+** | **+** | **-** | **-** |
| *Liver* | | | | | | | | | | |
| Glisson’s capsule | | **+** | | **+** | **+** | **+** | **+** | **+** | **+** | **+** |
| Hepato-cytes | Cytoplasm | **+** | | **+** | **+** | **+** | **+** | **+** | **+** | **+** |
|  | Nuclei | **-** | | **-** | **-** | **-** | **+** | **+** | **+** | **-** |
|  | Cell-cell junctions | **+** | | **+** | **+** | **-** | **-** | **-** | **-** | **-** |
| Kuppfer cells | | **+** | | **+** | **+** | **+** | **+** | **+** | **+** | **+** |
| Sinusoids | | **+** | | **+** | **+** | **+** | **-** | **+** | **+** | **-** |
| Connective tissue | | **+** | | **+** | **-** | **-** | **-** | **-** | **-** | **-** |
| Vascu-  lature | Smooth muscle cells | **+** | | **+** | **+** | **+** | **+** | **+** | **+** | **-** |
|  | Endothelial cells | **+** | | **+** | **+** | **+** | **+** | **+** | **+** | **-** |
| *Spleen* | | | | | | | | | | |
| Capsule | | **+** | | **+** | **+** | **+** | **+** | **+** | **+** | **+** |
| Red  Pulp | Cytoplasm | **+** | | **+** | **+** | **+** | **+** | **+** | **+** | **+** |
|  | Nuclei | **+** | | **+** | **-** | **-** | **+** | **+** | **+** | **-** |
| White  Pulp | T-cell area | **-** | | **-** | **-** | **-** | **-** | **-** | **-** | **-** |
|  | B-cell area | **+** | | **+** | **-** | **-** | **-** | **-** | **-** | **-** |
| Perivascular | | **+** | | **+** | **-** | **-** | **-** | **-** | **-** | **+** |
| Marginal zone | | **-** | | **+** | **-** | **-** | **-** | **-** | **-** | **+** |
| Trabeculae | | **+** | | **+** | **-** | **-** | **+** | **+** | **-** | **-** |
| Vascu-  lature | Smooth muscle cells | **+** | | **+** | **-** | **-** | **+** | **+** | **-** | **-** |
|  | Endothelial cells | **+** | | **+** | **+** | **+** | **+** | **+** | **-** | **-** |
| *Lung* | | | | | | | | | | |
| Pleura | | **+** | | **+** | **+** | **+** | **+** | **+** | **+** | **+** |
| Clara  Cells | Cytoplasm | **-** | | **+** | **+** | **+** | **+** | **+** | **+** | **+** |
|  | Nuclei | **-** | | **-** | **-** | **-** | **-** | **+** | **-** | **-** |
| Pneumocytes | | **+** | | **+** | **+** | **+** | **+** | **+** | **+** | **+** |
| Smooth muscle surrounding the bronchus | | **-** | | **+** | **-** | **-** | **-** | **+** | **-** | **-** |
| Connective tissue | | **+** | | **+** | **+** | **+** | **+** | **+** | **+** | **+** |
| Vascu-  lature | Smooth muscle cells | **-** | | **+** | **-** | **-** | **-** | **+** | **-** | **-** |
|  | Endothelial cells | **+** | | **+** | **+** | **+** | **+** | **+** | **+** | **+** |
